# Replicable and Coupled Changes in Innate and Adaptive Immune Gene Expression in Two Case-Control Studies of Blood Microarrays in Major Depressive Disorder

**DOI:** 10.1016/j.biopsych.2017.01.021

**Published:** 2018-01-01

**Authors:** Gwenaël G.R. Leday, Petra E. Vértes, Sylvia Richardson, Jonathan R. Greene, Tim Regan, Shahid Khan, Robbie Henderson, Tom C. Freeman, Carmine M. Pariante, Neil A. Harrison, MRC Immunopsychiatry Consortium, Edward Bullmore, Petra Vertes, Rudolf Cardinal, Sylvia Richardson, Gwenael Leday, Tom Freeman, Tim Regan, David Hume, Zhaozong Wu, Carmine Pariante, Annamaria Cattaneo, Patricia Zunszain, Alessandra Borsini, Robert Stewart, David Chandran, Livia Carvalho, Joshua Bell, Luis Souza-Teodoro, Hugh Perry, Neil Harrison, Wayne Drevets, Gayle Wittenberg, Declan Jones, Edward Bullmore, Shahid Khan, Annie Stylianou, Robbie Henderson, V. Hugh Perry, Wayne C. Drevets, Gayle M. Wittenberg, Edward T. Bullmore

**Affiliations:** a Medical Research Council Biostatistics Unit, Cambridge, United Kingdom; b Department of Psychiatry, University of Cambridge, Cambridge, United Kingdom; c Cambridgeshire and Peterborough National Health Service Foundation Trust, Cambridge, United Kingdom; d Roslin Institute, University of Edinburgh, Edinburgh, United Kingdom; e ImmunoPsychiatry, GlaxoSmithKline Research & Development, Stevenage, United Kingdom; f Institute of Psychiatry, Psychology and Neuroscience, King’s College London, London, United Kingdom; g Brighton and Sussex Medical School, University of Sussex, Brighton, United Kingdom; h Centre for Biological Sciences, University of Southampton, Southampton, United Kingdom; i Rancho BioSciences, San Diego, California; j Janssen Research & Development, Titusville, New Jersey

**Keywords:** Affymetrix, Bayesian, Biomarker, Inflammation, Systems, Transcriptome

## Abstract

**Background:**

Peripheral inflammation is often associated with major depressive disorder (MDD), and immunological biomarkers of depression remain a focus of investigation.

**Methods:**

We used microarray data on whole blood from two independent case-control studies of MDD: the GlaxoSmithKline–High-Throughput Disease-specific target Identification Program [GSK-HiTDiP] study (113 patients and 57 healthy control subjects) and the Janssen–Brain Resource Company study (94 patients and 100 control subjects). Genome-wide differential gene expression analysis (18,863 probes) resulted in a *p* value for each gene in each study. A Bayesian method identified the largest *p*-value threshold (*q* = .025) associated with twice the number of genes differentially expressed in both studies compared with the number of coincidental case-control differences expected by chance.

**Results:**

A total of 165 genes were differentially expressed in both studies with concordant direction of fold change. The 90 genes overexpressed (or UP genes) in MDD were significantly enriched for immune response to infection, were concentrated in a module of the gene coexpression network associated with innate immunity, and included clusters of genes with correlated expression in monocytes, monocyte-derived dendritic cells, and neutrophils. In contrast, the 75 genes underexpressed (or DOWN genes) in MDD were associated with the adaptive immune response and included clusters of genes with correlated expression in T cells, natural killer cells, and erythroblasts. Consistently, the MDD patients with overexpression of UP genes also had underexpression of DOWN genes (correlation > .70 in both studies).

**Conclusions:**

MDD was replicably associated with proinflammatory activation of the peripheral innate immune system, coupled with relative inactivation of the adaptive immune system, indicating the potential of transcriptional biomarkers for immunological stratification of patients with depression.

Depression and inflammation are often associated with one another. Depressive symptoms in a large population sample were significantly related to blood concentrations of C-reactive protein (CRP; odds ratio ∼1.8 for depressive symptoms in people with CRP > 3 mg/L vs. CRP < 1 mg/L) (1). Multiple case-control studies of major depressive disorder (MDD) have reported increased peripheral blood concentrations of CRP (Cohen’s *d* ∼0.50) and proinflammatory cytokines such as interleukin 6 (*d* ∼0.50) and tumor necrosis factor (*d* ∼0.40) in MDD 2, 3. The prevalence of comorbid depression is increased in many nonpsychiatric inflammatory disorders (4).

There is growing evidence for a causal effect of inflammation on depression. Peripheral inflammation precedes the emergence of depressive symptoms in longitudinal epidemiological studies (5) and in about 30% of patients receiving proinflammatory interferon-α treatment for hepatitis C 6, 7. Experimental challenge with peripheral proinflammatory stimuli in animals robustly induces a syndrome of illness behavior and anhedonia that approximates depressive symptoms (8). Peripheral immune cells and cytokines are known to mediate signals across the blood-brain barrier by several mechanisms (9). Activation of microglia can locally amplify the effects of even a weak peripheral proinflammatory signal on neuronal function and behavior (10).

These observations suggest that pharmacological disruption of peripheral proinflammatory signals could be therapeutically effective, at least for a subgroup of patients with depression. However, it is most unlikely that any single anti-inflammatory drug will prove to be superior to all existing treatments for all patients (11). Only about a third of patients with depression have biological evidence for peripheral inflammation [e.g., CRP > 3 mg/L (12)], and an anti-inflammatory drug seems likely a priori to be most effective for an inflamed subgroup of patients with depression. There are many markers of the peripheral immune system that can be conveniently measured in venous blood samples from patients with depression, including cytokines, CRP, and other proteins; cell counts from flow cytometry; and gene transcription (13).

Transcriptional (messenger RNA [mRNA]) biomarkers have the potential advantages of assay stability (compared with cytokines) and target specificity (compared with cell counts). However, previous case-control studies of peripheral blood gene expression and MDD have been inconsistent 14, 15, 16, 17, 18 (Supplemental Table S1). To date, the Netherlands Study of Depression and Anxiety (NESDA) (14) is the largest single case-control study of the peripheral blood transcriptome in MDD (882 current patients with MDD, 635 remitted patients with MDD, and 331 control subjects). The NESDA reported statistically significant (false discovery rate [FDR] = 10%) differential expression of 129 genes enriched for interleukin 6 and natural killer (NK) cell signaling pathways (19).

We were primarily motivated by the hypothesis that MDD is associated with peripheral blood transcriptional markers of innate immune system activation 8, 20. We were also concerned to focus on results that were more likely to replicate across case-control studies of gene expression in MDD. We report Affymetrix microarray data on 18,863 probes from two independently designed and conducted case-control studies of MDD. We used a Bayesian method to identify genes that were differentially expressed in both studies. Focusing on a consensus set of 165 genes, we investigated the functional significance of the genes that were differentially (over- or under-) expressed in cases compared with controls. We also explored the secondary hypothesis that innate immune system activation is coupled to relative inactivation of the adaptive immune system in patients with MDD 21, 22, 23.

## Methods and Materials

### Samples

We analyzed data from two case-control studies of depression: the GlaxoSmithKline–High-Throughput Disease-specific target Identification Program (GSK–HiTDiP) study and the Janssen–Brain Resource Company (Janssen–BRC) study. Other aspects of these studies have been previously reported 24, 25, 26, 27, 28; demographic and clinical details on the samples are provided in Supplemental Table S2.

#### GSK–HiTDiP

This study was designed primarily to test for an association between genetic (DNA) variation and diagnosis of depression. Minimal sociodemographic and clinical data were collected, but microarray data were available for analysis (after quality control) from whole-blood samples stored for less than 6 years on a sample comprising 113 patients with MDD prospectively balanced for comorbid anxiety disorder [57 with generalized anxiety disorder diagnosed by the Mini-International Neuropsychiatric Interview (29) and 56 without anxiety disorder] and 57 healthy control subjects. All participants provided informed consent in writing. The study was approved by an independent ethics review board.

#### Janssen–BRC

This study was designed primarily for biomarker discovery. Microarray data were available for analysis (after quality control) from whole-blood samples stored for less than 1 year on a sample comprising 94 patients with MDD (40 with generalized anxiety disorder diagnosed post hoc by the Mini-International Neuropsychiatric Interview and 54 without anxiety disorder) and 100 healthy control subjects. Additional data on melancholic symptom severity, anxiety, substance use, and body mass index (BMI) were available for patients with MDD. All participants provided informed consent in writing. The study was approved by an independent ethics review board.

Whole-blood samples from both studies were analyzed using the Affymetrix Human Genome U133 plus 2.0 array. We applied identical quality control, normalizing, and annotation algorithms to the two datasets, resulting in the estimation of mRNA expression at each of 18,863 unique probes for each participant.

### Differential Expression Analysis

To determine differential gene expression between cases and controls, we adopted the same strategy for both studies; see Supplemental Figure S1 for a schematic overview of the data analysis strategy. For each gene *i* = 1, …, 18,863, we fitted a linear regression model that included group (coded Gr; two-level factor, case/control), batch (B; two-level factor, 1/2), gender (Ge; two-level factor, male/female), age (Ag; continuous), and presence/absence of anxiety (An; two-level factor, 0/1) as covariates. Denoting samples by *j* = 1, …, *n* and the design matrix by *X*, the model is

(1) $$y_{ij}=\beta_{i0}+\sum_{k=1}^{2}\beta_{ik}^{Gr}x_{jk}^{Gr}+\sum_{l=1}^{2}\beta_{il}^{B}x_{jl}^{B}+\sum_{m=1}^{2}\beta_{im}^{Ge}x_{jm}^{Ge}+\beta_{i}^{Ag}x_{j}^{Ag}+\sum_{r=1}^{2}\beta_{ir}^{An}x_{jr}^{An}+\varepsilon_{i},$$

where *ε*_*i*_ ∼ *N*(0, *σ*_*i*_^2^). For identifiability, we imposed the contrasts $\beta_{i1}^{Gr}=\beta_{i1}^{B}=\beta_{i1}^{Ge}=\beta_{i1}^{An}=0$ on model parameters. The model was fitted using the R package limma (30). Subsequently, we tested the null hypothesis *H*_i0_: *β*_*i*2_^*Gr*^ = 0; that is, there is no difference in expression of the *i*th gene between the two groups using the moderated *t* statistic (31). For each of the 18,863 probes in each study, the *p* value was generated using the asymptotic approximate distribution (and also nonparametrically by a permutation algorithm; see Supplemental Tables S3 and S4).

### Combining *p* Values for Differential Gene Expression From Two Studies

To identify MDD-related genes that were replicated in both the GSK–HiTDiP and Janssen–BRC datasets, we set the *p*-value threshold for significance of differential expression of each gene in each study to optimize in some sense the number of genes that were differentially expressed in both studies compared with the number of coincidental differences expected by chance. To do this, we used the method of Blangiardo and Richardson (32), implemented in the R package sdef (33), and specified the *p*-value threshold as $q_{2}$, which represents the largest (most lenient) *p* value < .05 for which there are at least twice as many significant case-control differences in common between the two studies as expected by chance.

### Gene Coexpression Network Analysis

We used weighted gene correlation network analysis (34) to construct a normative coexpression network in which nodes represent genes and weighted edges represent correlations between the expression of pairs of genes in healthy control subjects (34). To maximize the amount of data available for this estimation, and to ensure that the resulting network was representative of both studies, we included the healthy control data from both the GSK–HiTDiP and Janssen–BRC studies (*N* = 157 in total). We used the consensus weighted gene correlation network analysis method to construct a weighted undirected graph that could be decomposed into a set of modules of strongly coexpressed genes (34). Eigengenes were used to test for case-control differences in the gene expression profile on average within each module of the normative weighted gene correlation network analysis transcriptome (Supplemental Table S11).

### Gene Ontology Enrichment Analysis

We performed enrichment analysis for various gene lists such as the list of genes differentially expressed in both studies and the list of genes comprising each module of the coexpression network. We used the R package topGO (35) to determine whether these lists were significantly enriched for specific Gene Ontology (GO) terms (36) using a stringent Bonferroni correction (*q* < .05) for multiple comparisons across all 10,124 terms, resulting in a *p*-value threshold of 4.94 × 10^−6^.

### Protein–Protein Interaction Mapping

We used the Search Tool for the Retrieval of Interacting Genes/Proteins [http://string-db.org (37)] to determine the network of known protein–protein interactions among the genes differentially expressed in both studies (38).

### Cell Type–Specific Gene Expression Analysis

We investigated the genes differentially expressed in both studies in relation to a third independent microarray (Affymetrix) dataset designed to examine cell type–specific expression patterns (39). In particular, we focused on the expression of MDD-related genes across 37 sorted cell samples of the following immune cell types: erythroblasts (8 samples), monocytes (6 samples), monocyte-derived dendritic cells (5 samples), neutrophils (3 samples), B cells (4 samples), CD4+ T cells (5 samples), CD8+ T cells (5 samples), and NK cells (6 samples). We used the BioLayout Express^3D^ software 40, 41 to visualize clusters or subgroups of genes that shared similar patterns of expression across different cell types.

## Results

### Combining Differential Expression Across Two Case-Control Studies of MDD

Separate differential expression analyses of the GSK–HiTDiP and Janssen–BRC datasets yielded two lists of *p* values for the same set of 18,863 gene expression probes (Supplemental Tables S3 and S4). The Bayesian method identified *q*_2_ = .0246 as the largest *p* value threshold associated with twice the number of genes differentially expressed in both studies compared with the number of coincidental case-control differences expected by chance. At this threshold, 173 genes were differentially expressed in both studies. We further refined this gene list by restricting attention to the 165 consensus genes (95%) that showed the same direction of overexpression (UP) or underexpression (DOWN) in the two case-control studies (Supplemental Table S5).

These findings were corroborated by Fisher’s chi-square test for combining *p* values, which identified 393 genes that were differentially expressed in both studies with FDR = 10%, of which 146 were included in the list of replicably, concordantly, differentially expressed genes defined by Bayesian analysis (Supplemental Table S6).

Furthermore, the set of 165 replicably concordant genes (henceforth the MDD-165 consensus set) was partially corroborated by the prior results of the NESDA case-control study (14). Considering the 15,830 genes that were measured by the microarrays used in all three studies (NESDA, Janssen–BRC, and GSK–HiTDiP), 150 of the MDD-165 consensus set were included, of which 7 genes (*CD247, PRKCH, PGLYRP1, NFATC2, ST6GAL1, MAPK14,* and *MTSS1*) were also differentially expressed, with the same sign of fold change, in the NESDA study at FDR = 5%. When the FDR threshold for the NESDA study was relaxed to 10% and 20%, 18 and 45 of the 150 genes differentially expressed in both the GSK–HiTDiP and Janssen–BRC studies, respectively, were also differentially expressed in the NESDA study (Supplemental Table S5). By comparison, we expect by chance to find 1.95, 8.30, and 30.40 genes in common with the NESDA study at FDR thresholds of 5%, 10%, and 20%, respectively. In contrast, there was less overlap between the MDD-165 consensus set and the top 29 most significantly differentially expressed genes reported in another large case-control study of MDD (16); only *SRSF5* was underexpressed in both of these lists.

To assess the robustness of our results to key modeling assumptions, we conducted two sensitivity analyses. First, we repeated the analysis using a model for differential expression that did not include comorbid anxiety as an explanatory factor. Only 24 genes were identified by Bayesian analysis as significantly coexpressed; of these 24 genes, 21 were concordant for sign of fold change, and 15 of these 21 genes were also included in the MDD-165 consensus set (Supplement). The reduced sensitivity when not adjusting for anxiety likely reflects the importance of refining or homogenizing the clinical phenotype in the search for biomarkers. Second, we repeated the analysis using a model for differential expression that included BMI as a covariate. BMI was not measured in the GSK–HiTDiP study, so this analysis was restricted to 74 patients and 80 control subjects from the Janssen–BRC dataset. Inclusion of BMI in the model tended to increase the *p* values for MDD-related differences, and only 73 genes in the MDD-165 consensus set were differentially expressed at the probability threshold *q*_2_ = .0246 (Supplemental Figure S3). The reduced sensitivity of the analysis including BMI as a covariate likely reflects the known proinflammatory effect of obesity and the reduced sample size available.

### Gene Set Enrichment Analyses Across GO Terms

We conducted a series of enrichment analyses to functionally characterize the MDD-165 consensus set. The top 10 most significantly enriched GO terms all were related to the immune response to infection (Bonferroni *q* < .05 for the top 9 terms) (Supplemental Table S7). Among the MDD-165 consensus set, 90 genes were overexpressed in cases compared with controls. The UP gene list was significantly enriched for GO terms related to the innate immune system and response to infection (Bonferroni *q* < .05 for the top 17 terms) (Table 1). In addition, 75 genes were significantly underexpressed in cases compared with controls. As we hypothesized based on prior literature (19), this gene list was enriched for GO terms related to the adaptive immune system (*p* ≤ 10^−3^ for top 20 GO terms; Table 1), but there was no significant enrichment of the DOWN genes at the more stringent Bonferroni threshold.

**Table 1:** Enrichment Analysis of the MDD-165 Consensus Set of Genes That Were Differentially Overexpressed (UP) or Underexpressed (DOWN) in Patients With MDD Compared With Healthy Control Subjects in Both GSK–HiTDiP and Janssen–BRC Studies

Rank	GO:ID	Term	Annotated	Found	Expected	Fisher’s Test
Overexpressed UP (Innate Immunity)						
1	GO:0009617	Response to bacterium	405	19	2.29	7.7e-13
2	GO:0042742	Defense response to bacterium	164	13	0.93	7.0e-12
3	GO:0043207	Response to external biotic stimulus	651	20	3.69	3.7e-10
4	GO:0051707	Response to other organism	651	20	3.69	3.7e-10
5	GO:0009607	Response to biotic stimulus	680	20	3.85	8.0e-10
6	GO:0002376	Immune system process	2149	35	12.17	9.8e-10
7	GO:0006955	Immune response	1310	26	7.42	6.3e-09
8	GO:0098542	Defense response to other organism	351	14	1.99	9.1e-09
9	GO:0051704	Multi-organism process	2176	32	12.32	9.2e-08
10	GO:0050832	Defense response to fungus	21	5	0.12	9.7e-08
11	GO:0032496	Response to lipopolysaccharide	253	11	1.43	1.7e-07
12	GO:0002237	Response to molecule of bacterial origin	265	11	1.5	2.8e-07
13	GO:0006952	Defense response	1372	24	7.77	3.2e-07
14	GO:0009605	Response to external stimulus	1947	29	11.02	3.8e-07
15	GO:0045087	Innate immune response	820	18	4.64	5.5e-07
16	GO:0009620	Response to fungus	34	5	0.19	1.3e-06
17	GO:0019731	Antibacterial humoral response	23	4	0.13	7.8e-06
18	GO:0031640	Killing of cells of other organism	25	4	0.14	1.1e-05
19	GO:0044364	Disruption of cells of other organism	25	4	0.14	1.1e-05
20	GO:0019730	Antimicrobial humoral response	27	4	0.15	1.5e-05
Underexpressed DOWN (Adaptive Immunity)						
1	GO:0050851	Antigen receptor-mediated signaling path	133	5	0.59	0.00029
2	GO:0046649	Lymphocyte activation	532	9	2.34	0.0005
3	GO:0009059	Macromolecule biosynthetic process	4336	32	19.09	0.00052
4	GO:0034645	Cellular macromolecule biosynthetic proc	4183	31	18.42	0.00064
5	GO:0010557	Positive regulation of macromolecule bio	1361	15	5.99	0.00066
6	GO:0032774	RNA biosynthetic process	3275	26	14.42	0.00086
7	GO:0046631	Alpha-beta T cell activation	99	4	0.44	0.00094
8	GO:0016070	RNA metabolic process	3873	29	17.06	0.00095
9	GO:0050776	Regulation of immune response	721	10	3.18	0.00112
10	GO:0006351	Transcription, DNA-templated	3161	25	13.92	0.00122
11	GO:0097659	Nucleic acid-templated transcription	3177	25	13.99	0.00131
12	GO:0042110	T cell activation	382	7	1.68	0.0014
13	GO:0070489	T cell aggregation	382	7	1.68	0.0014
14	GO:0071593	Lymphocyte aggregation	384	7	1.69	0.00144
15	GO:0031328	Positive regulation of cellular biosyn	1470	15	6.47	0.00147
16	GO:0002562	Somatic diversification of immune recept	52	3	0.23	0.00154
17	GO:0016444	Somatic cell DNA recombination	52	3	0.23	0.00154
18	GO:0070486	Leukocyte aggregation	390	7	1.72	0.00158
19	GO:0045893	Positive regulation of transcription, DN	1178	13	5.19	0.00161
20	GO:1903508	Positive regulation of nucleic acid-temp	1178	13	5.19	0.00161

The top 20 GO terms for biological processes are ranked according to their *p* values by Fisher’s exact test for significant enrichment. Bonferroni correction specifies a *p*-value threshold of *p* = 4.94 × 10^−6^ to achieve significance at *q* < 0.05. Such a correction is well known to be too stringent given that many of the GO terms are correlated; however, the top 17 terms were significantly enriched in the overexpressed UP gene set after Bonferroni correction.

GO, Gene Ontology; GSK–HiTDiP, GlaxoSmithKline–High-Throughput Disease-specific target Identification Program; Janssen–BRC, Janssen–Brain Resource Company; MDD, major depressive disorder; MDD-165 consensus set, set of 165 replicably concordant genes.

### Protein Interaction and Whole-Genome Transcriptional Network Analyses

**Figure 1:**
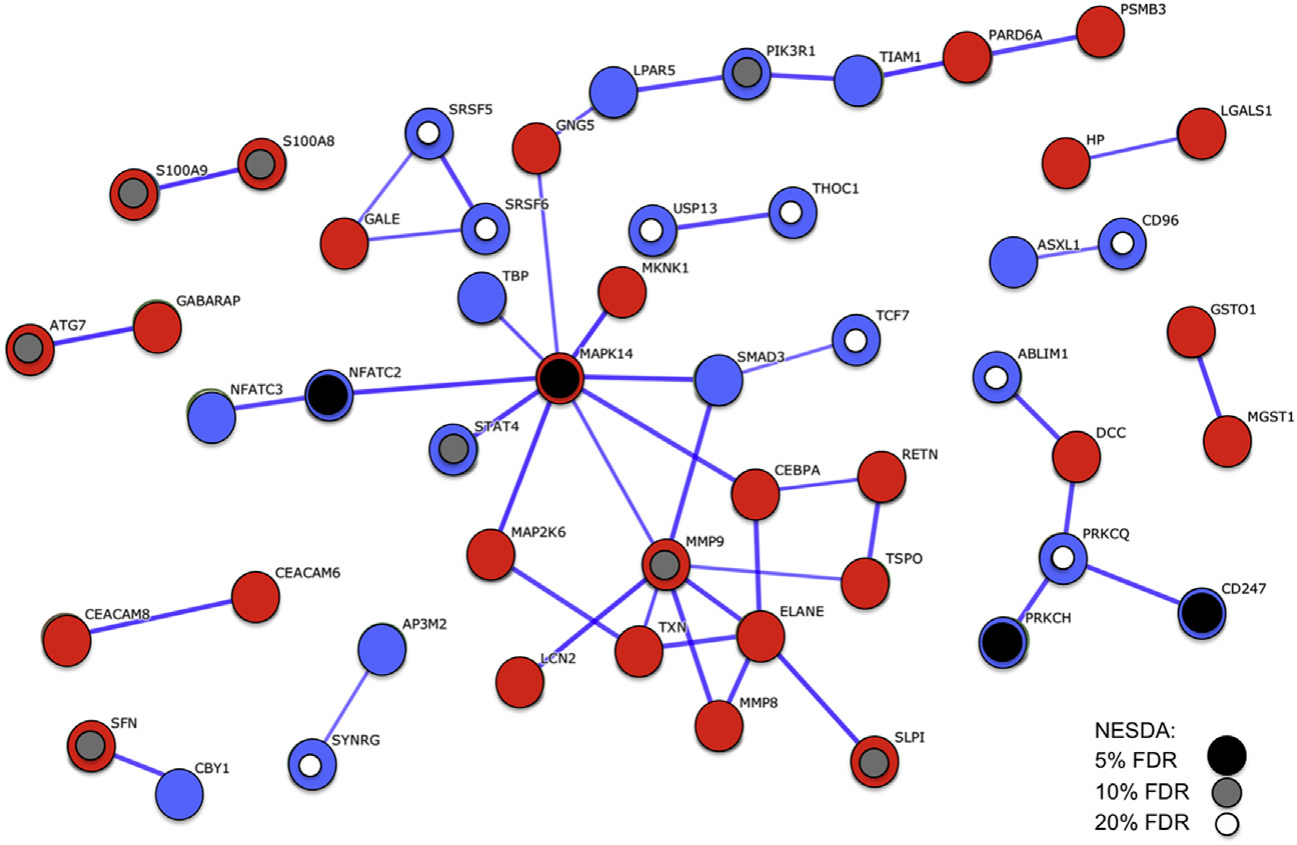
Protein–protein interaction network for proteins coded by the set of 165 replicably concordant genes differentially expressed in both case-control studies of major depressive disorder. The network is represented by an undirected graph where links correspond to known protein–protein interactions and weights are proportional to the Search Tool for the Retrieval of Interacting Genes/Proteins confidence score (38). Only high-confidence (>0.7) links are retained, and disconnected genes are not shown. Red (blue) nodes correspond to genes over- (under-) expressed in major depressive disorder in both the GlaxoSmithKline–High-Throughput Disease-specific target Identification Program and Janssen–Brain Resource Company datasets. Smaller inner circles highlight proteins that are coded by genes also differentially expressed and with the same sign of fold change in a third large independent case-control study of major depressive disorder (Netherlands Study of Depression and Anxiety [NESDA]) (14) thresholded to control false discovery rate (FDR) at 20% (white circles), 10% (gray circles), and 5% (black circles).

We used Search Tool for the Retrieval of Interacting Genes/Proteins analysis (37) to visualize the network of protein–protein interactions between the proteins coded by the MDD-165 consensus set. These genes were significantly enriched for protein–protein interactions (permutation test, *p* = 7 × 10^−4^) that were concentrated around mitogen-activated protein kinase 14 (MAPK14) and matrix metalloproteinase 9 (MMP9), which thus can be regarded as the most highly interactive hubs of this immune signaling network (Figure 1). Many of the proteins in this network are coded by genes that were independently identified by the NESDA study as differentially expressed in MDD; see Figure 1.

**Figure 2:**
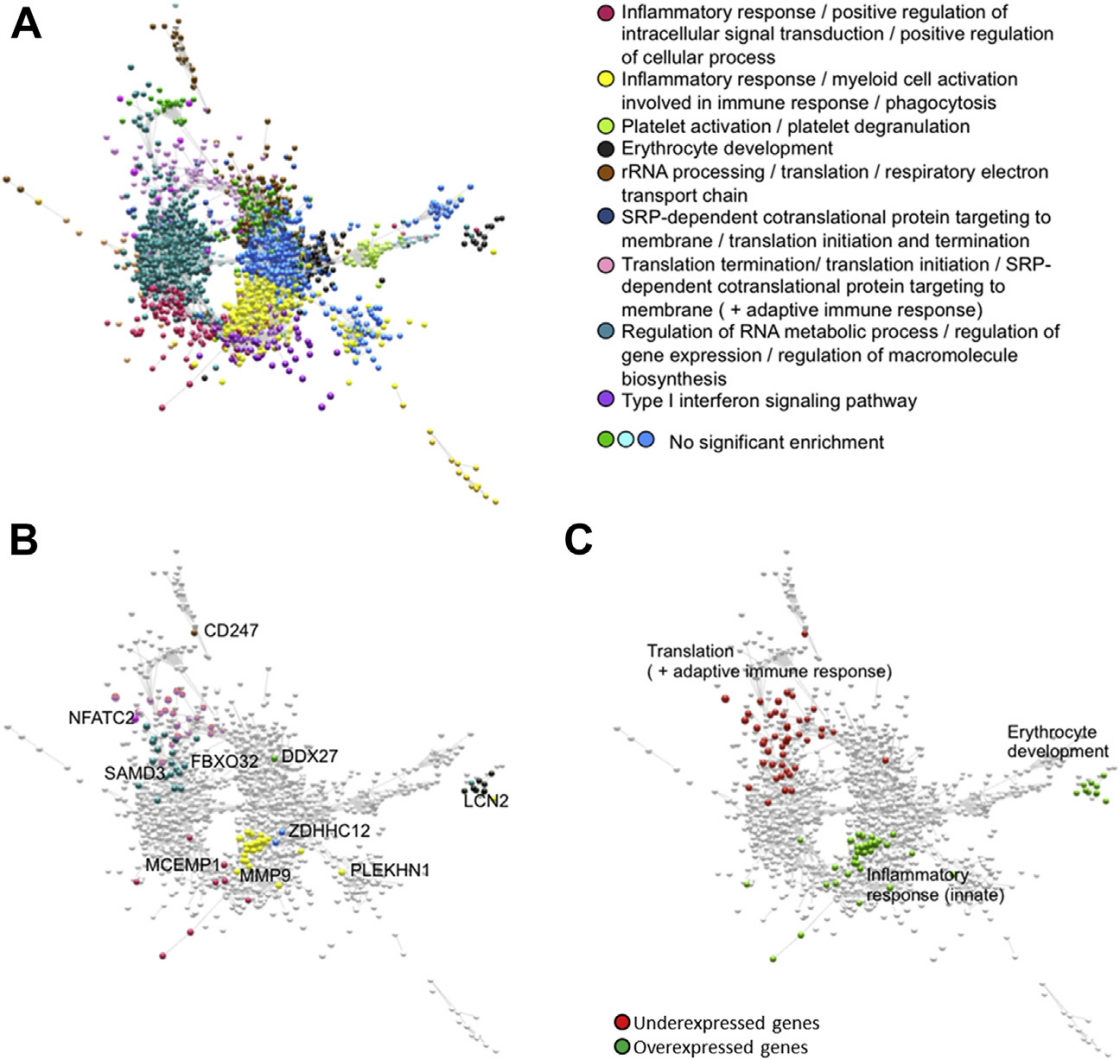
Major depressive disorder (MDD)-related genes in the context of the normative whole-genome transcriptome. Overexpressed genes (or UP genes) in patients with MDD are concentrated in a module of the normative gene coexpression network specialized for innate immune response, whereas underexpressed genes (or DOWN genes) are concentrated in a module partially specialized for adaptive immune response. **(A)** The modules of the normative transcriptome are highlighted in different colors. **(B)** The MDD-related genes are colored according to their normative module affiliation, and representative genes are text-labeled. **(C)** The MDD-related genes are colored green for overexpressed (or UP) genes and are colored red for underexpressed (or DOWN) genes. The text labels highlight the functions of the corresponding modules of the normative transcriptome. rRNA, ribosomal RNA; SRP, signal recognition particle.

We constructed a graph representing significant coexpression of a pair of genes (nodes) as an edge drawn between them. As previously reported (42), this whole-genome transcriptional network or transcriptome had complex topological properties, including a community structure comprising modules of coexpressed genes enriched for specific GO terms (Figure 2). The UP genes were concentrated in the normative module (red) significantly enriched for innate immune response GO terms (e.g., myeloid cell activation involved in immune response, *q* = 5 × 10^−5^, Bonferroni corrected), whereas the DOWN genes were concentrated in a normative module (pink) specialized for translation-based terms (Figure 2). We confirmed in a hypothesis-driven analysis that the module associated with DOWN gene expression was also significantly enriched for adaptive immune response terms (e.g., T-cell differentiation, *p* = .0002). There were significant case-control differences in eigengene scores summarizing expression of all genes within modules enriched for innate immune response (red, *p* = .031; yellow, *p* = .015), translation (pink, *p* = .002), and one additional module with no significant enrichment terms (tan, *p* = .025); see Supplemental Table S11.

### Cell Type–Specific Expression Patterns for the MDD-165 Consensus Set

**Figure 3:**
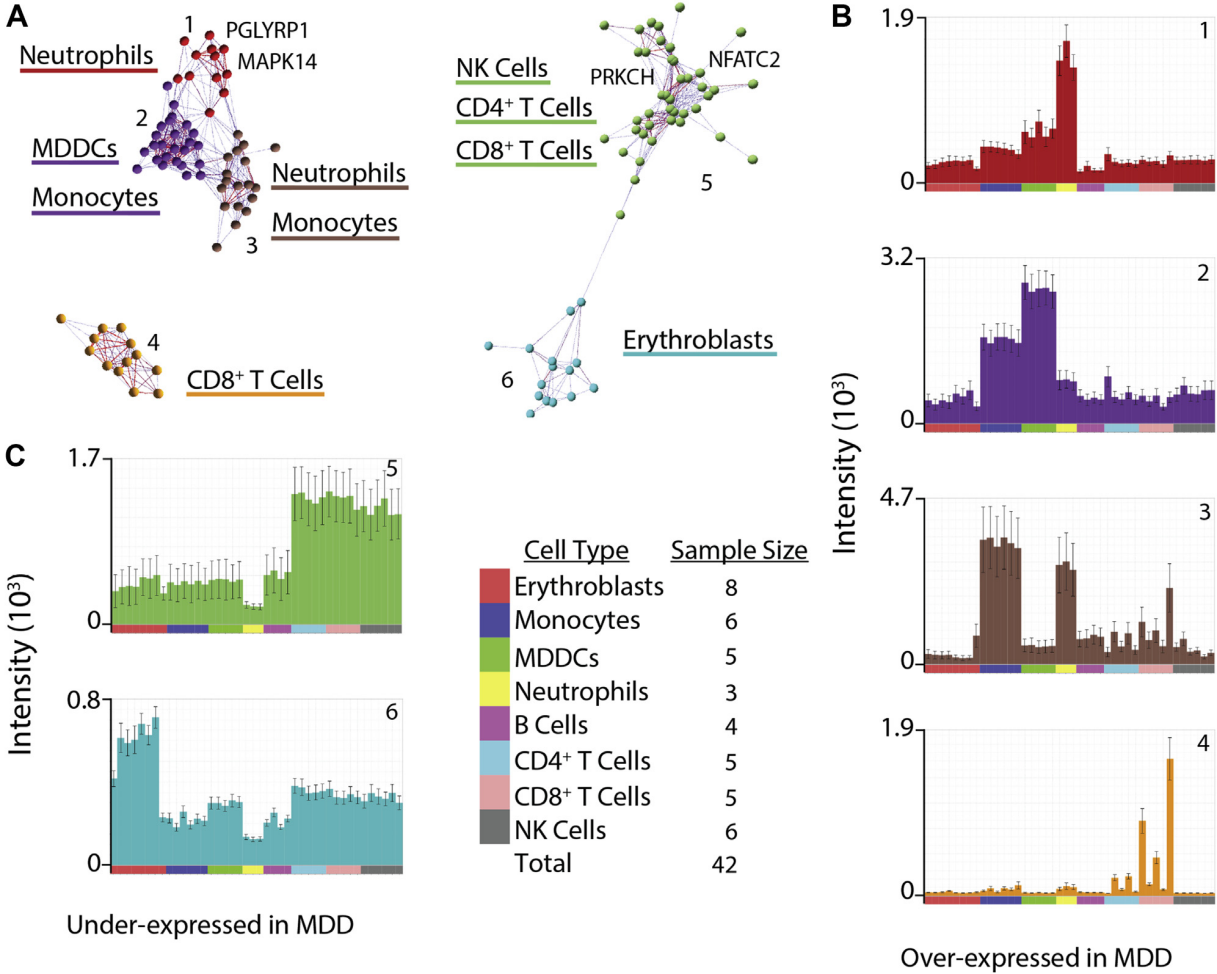
Cell class–specific expression patterns for the set of 165 replicably concordant genes. We estimated the correlation between each possible pair of the 165 major depressive disorder (MDD)-related genes and identified clusters of genes with similar expression patterns in an independent microarray dataset on specific cell types. The set of 165 replicably concordant genes formed six clusters, with each cluster comprising a subset of genes that had strong mutual coexpression across a range of eight distinct cell classes: erythroblasts, monocytes, monocyte-derived dendritic cells (MDDCs), neutrophils, B cells, CD4^+^ T cells, CD8^+^ T cells, and natural killer (NK) cells. **(A)** The six clusters of genes with strongly correlated expression profiles. Clusters 1 to 4 (left) comprised genes that were overexpressed in MDD (or UP genes), and clusters 5 and 6 (right) comprised genes that were underexpressed in MDD (or DOWN genes). **(B)** Histograms of clustered gene expression across cell types for each of the four clusters of UP genes overexpressed in MDD (from top to bottom: clusters 1–4). The x-axis color legend codes for different cell types. **(C)** Histograms of clustered gene expression across cell types for each of the two clusters of DOWN genes underexpressed in MDD (from top to bottom: clusters 5 and 6). The x-axis color legend codes for different cell types.

To assess the cellular specificity of the MDD-165 consensus set, we used microarray data from an independent study of cell type–specific gene expression across eight major classes of immune cells in healthy control subjects (39). Remarkably, 89 of the 90 UP genes formed four clusters of gene coexpression, three of which represented strong overexpression of a subset of UP genes in one or two myeloid cell classes (Figure 3). Likewise, 71 of the 75 DOWN genes formed two clusters of gene coexpression, each representing cell-specific overexpression of a subset of DOWN genes in one or two lymphoid or erythroblast classes (Figure 3).

### Coupled UP and DOWN Gene Expression in Patients With MDD

**Figure 4:**
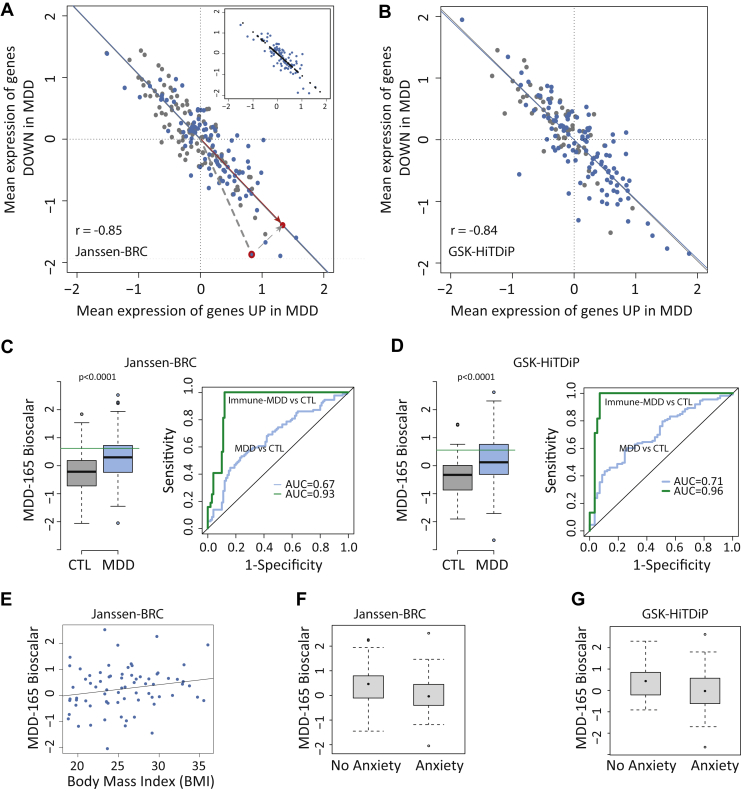
Opposing and coordinated expression of innate and adaptive immune transcripts in patients with major depressive disorder (MDD). **(A, B)** Scatter plots for the mean expression across the 75 underexpressed (DOWN) genes plotted against the mean expression of 90 overexpressed (UP) genes in the Janssen–Brain Resource Company (Janssen–BRC) **(A)** and GlaxoSmithKline–High-Throughput Disease-specific target Identification Program (GSK–HiTDiP) **(B)** datasets. Each point corresponds to a patient. Blue indicates case, and gray indicates control (CTL). Regression lines are shown in blue and gray, respectively. In **(A)**, an individual patient’s data point (red outline) is projected onto the regression line (red circle). The distance from the origin to the point on the regression line is the bioscalar value for that patient. Inset illustrates the projection (black) of all individual patient data points (blue) onto the sample regression line. **(C, D)** Left panels: Box plot of MDD-165 bioscalar values in controls and cases. Green line indicates the threshold identifying the top third (tertile) of the MDD-165 bioscalar distribution as a subgroup of inflamed patients with MDD (designated immune-MDD). Right panels: Receiver operating characteristic curves were calculated for MDD vs. CTL and immune-MDD vs. CTL classifications. **(E)** Correlation of each depressed patient’s MDD-165 bioscalar with body mass index (BMI). **(F, G)** Box plots of MDD-165 bioscalar for subgroups defined by a comorbid diagnosis of anxiety disorder. AUC, area under the curve.

To explore the extent to which UP gene overexpression was related to DOWN gene underexpression, we estimated the mean UP gene expression (over all 90 differentially overexpressed genes) and the mean DOWN gene expression (over all 75 differentially underexpressed genes) for each of 113 patients with MDD in the GSK–HiTDiP study and each of 94 patients with MDD in the Janssen–BRC study. In both studies, UP and DOWN expression scores were strongly negatively correlated (Figure 4), indicating that these case-control differences were coupled at the level of individual patients (Janssen–BRC: *r* = −.82, *p* < 10^−4^; GSK–HiTDiP: *r* = −.74, *p* < 10^−4^).

Given the strong linear relationship between UP and DOWN gene expression in patients with MDD, we mapped each patient’s data to a point on the fitted regression line, thereby characterizing each patient by a bioscalar that locates it on a single axis or dimension of coupled UP/DOWN gene expression; see Figure 4A (inset). As expected, there were significant case-control differences in the MDD-165 bioscalar in both studies (Cohen’s *d* = 0.68 for GSK–HiTDiP and 0.58 for Janssen–BRC, corresponding to a medium effect size; Figure 4C, D), with patients on average having more positive values, indicating a greater shift in the direction of coupled innate activation and adaptive inactivation. Receiver operating characteristic analysis indicated that cases and controls were classified with an area under the curve of 0.71 for the GSK–HiTDiP study and an area under the curve of 0.67 for the Janssen–BRC study (Figure 4C, D). This moderately accurate classification performance reflects the fact that the bioscalar distributions of cases and controls are overlapping. We also assessed the performance of the bioscalar not to discriminate cases from controls (which is ultimately a clinical diagnostic decision) but rather to identify the top third “most inflamed” patients with MDD. To do this, we defined a cutoff value for the bioscalar corresponding to the top tertile critical value of its distribution in each study (0.57). Participants with MDD-165 > 0.57 were classified correctly as belonging to such an inflamed MDD subgroup (sensitivity = 100% by definition) with a high specificity (93% and 88% in GSK–HiTDiP and Janssen–BRC studies, respectively).

We provisionally explored correlations between the MDD-165 bioscalar and sociodemographic or clinical differences among the patients with MDD in the Janssen–BRC study. BMI (*r*_72_ = .20, *p* = .099), the CORE total score for melancholic symptom severity (*r*_88_ = .18, *p* = .097), and the presence or absence of comorbid substance abuse disorder (two-tailed *t*_63_ = 1.9, *p* = .07) were not significantly associated with bioscalar scores. There was a significant difference in bioscalar scores between subgroups of patients with MDD with or without comorbid anxiety disorder (*t*_83_ = −2.4, *p* = .02).

## Discussion

We have reported the differential expression analysis of whole-genome microarray data from two independent case-control studies of patients with MDD compared with healthy control subjects. Using Bayesian methods, we identified a set of 165 consensus genes that were replicably associated with MDD, sharing the same direction of fold change in the cases compared with controls in both studies. The robustness of these results was further supported by comparison with the results of the largest prior case-control microarray study of depression, the NESDA study (14); several genes differentially expressed in the NESDA study were likewise differentially expressed in these data, for example, *MAPK14* and *MMP9.* Both of these genes were consistently overexpressed in patients with MDD and both code proteins (MAPK14 and matrix metallopeptidase 9) that were hubs of a network of interactions between immune signaling proteins coded by many of the other differentially expressed genes. A MAPK14 inhibitor has been tried for treatment of major depression but did not demonstrate consistently significant effects on symptom rating scales compared with placebo at the single dose tested (43).

The consensus set of 165 genes associated with MDD was divided into two approximately equal-sized subsets; here, 90 so-called UP genes were overexpressed in patients and 75 so-called DOWN genes were underexpressed in patients. The overexpressed UP genes were significantly enriched for GO terms related to the response to infection and the innate immune system. These gene transcripts were not functionally unrelated to or independent of each other. Most UP genes were affiliated with the module of the normative gene transcriptional network specialized for innate immune response. Smaller clusters of highly correlated UP genes were typically enriched in neutrophils, monocytes, and monocyte-derived dendritic cells. These results are compatible with prior data implicating activation of the innate immune system and increased proinflammatory cytokine signaling in (some people with) depression 8, 20.

In contrast, we found that the underexpressed DOWN genes were enriched for GO terms related to T-cell function and adaptive immunity. The DOWN genes were affiliated with modules of the transcriptome partially specialized for adaptive immune function, and smaller clusters of strongly coexpressed DOWN genes were enriched in T cells, B cells, and NK cells. These results are compatible with prior data implicating relative suppression of the cellular immune system in (some people with) depression, including evidence for decreased NK cell cytotoxicity and decreased proliferation of lymphocytes challenged with mitogens in vitro (44), and with recent data suggesting that deficiencies of NK and T cells co-occur with inflammatory monocyte activation as related phenomena in the same patients with MDD 21, 22, 45, 46.

In both studies, we also observed a strong statistical association between mean UP gene overexpression and mean DOWN gene underexpression in each patient. This provided robust evidence that these complementary immunophenotypes are indeed coupled at the level of an individual patient. It also motivated the concept that each individual patient may be located by a single number (bioscalar) on a spectrum of coupled change in innate and adaptive immune system function. Patients with MDD had significantly higher scores on this bioscalar, indicating a mean shift to relatively increased UP gene expression and decreased DOWN gene expression. The subgroup of most-inflamed patients with MDD, defined as the top tertile of the MDD-165 bioscalar distribution, were identified with high sensitivity and specificity, suggesting that this may in the future prove to be a useful biomarker for defining an abnormally inflamed subgroup of patients with MDD.

So far, we have used the terms overexpression and underexpression simply to describe higher and lower levels of measured mRNA in cases compared with controls. At least three (mutually nonexclusive) explanatory factors are plausible: cellular, genetic, and environmental. First, case-control differences in expression could reflect differences in cell counts. For example, increased monocyte counts have been reported in MDD (47) and could cause apparent overexpression of innate immune system genes measured in whole-blood samples (and relative underexpression of adaptive immune genes). It will be important in the future to combine cytometry and transcriptional measurements in the same patients and to measure case-control expression differences in sorted cell samples. Second, mRNA changes could be quantitative traits determined by DNA variation at expression quantitative trait loci. This genetic explanation would require that (at least some) patients with MDD had a consistent profile of allelic variation in inflammation-related genes (48), but large genome-wide association studies have so far failed to identify genetic variants robustly linked to risk for major depression (49). The absence of significant genome-wide association study findings in MDD could be regarded as problematic for an expression quantitative trait locus interpretation of expression changes, or it could be discounted on the grounds that classically designed association studies were underpowered to detect DNA variations occurring in only a subgroup of immunologically dysfunctional patients. Third, gene expression changes could have been induced in patients by exposure to some shared environmental stimulus. The risk for depression is associated with adverse events in the biological and social environments such as infection, childhood abuse, and bereavement (50). In addition, psychosocial stress has been linked to peripheral immune state changes, such as increased proinflammatory cytokines and monocyte activation (51), that are compatible with overexpression of innate immune system genes.

It is a methodological limitation that the GSK–HiTDiP study, designed primarily to identify risk genes for MDD, did not provide data on severity of depressive symptoms or BMI. The case-control comparisons are not controlled for potentially confounding effects of cigarette smoking, race, comorbid medical disease, or socioeconomic status on peripheral immune status. Future studies of transcriptional biomarkers in MDD, with more detailed clinical and immunological phenotyping and more complete control of potential confounds, will be required to evaluate the generalizability of these results.

In short, we have reported replicable new evidence in support of peripheral immune gene expression markers for MDD. It may be fruitful in the future to investigate coupled overexpression of innate immune genes and underexpression of adaptive immune genes as a predictor of antidepressant response to novel immunotherapeutics.
