# Supplementary material for: Replicable and Coupled Changes in Innate and Adaptive Immune Gene Expression in Two Case-Control Studies of Blood Microarrays in Major Depressive Disorder
Source: Biol Psychiatry. 2018 Jan 1;83(1):70–80. doi: 10.1016/j.biopsych.2017.01.021 (PMC5720346; doi:10.1016/j.biopsych.2017.01.021)
Supplement: Supplemental Material [file mmc1.pdf]

Replicable and Coupled Changes in Innate and Adaptive Immune Gene Expression in Two Case-Control Studies of Blood Microarrays in Major Depressive Disorder

Supplemental Information

Contents

List of MRC Immunopsychiatry Consortium Members

Supplementary Text

Samples

Microarray data

Differential gene expression in each case-control study separately

Combining P-values for differential gene expression from two studies

Weighted gene correlation network analysis

Effects of controlling for comorbid anxiety

Supplementary Tables

Table S1. Summary of case-control studies of whole genome expression in peripheral blood cells from patients with major depressive disorder compared to healthy controls.

Table S2. Patient demographics in Janssen-BRC and GSK-HiTDiP data sets.

Table S3. List of differentially expressed genes in the GSK-HiTDiP data set.

Table S4. List of differentially expressed genes in the Janssen-BRC data set.

Table S5. List of 165 genes that are significantly differentially expressed with consistent sign of fold change in both the GSK-HiTDiP and Janssen-BRC studies.

Table S6. List of 393 genes that are significantly differentially expressed across both studies using Fisher’s chi squared test with FDR=10%.

Table S7. Enrichment analysis of genes differentially expressed in both GSK-HiTDiP and Janssen-BRC studies.

Table S8. List of 150 genes that overlap with the NESDA study.

Table S9. List of 21 genes that are significantly differentially expressed with consistent sign of fold change in both the GSK-HiTDIP and Janssen-BRC studies when not correcting for anxiety.

Table S10. Enrichment analysis of the MDD-21 consensus set of genes that were differentially over-expressed or under-expressed in MDD patients compared to healthy controls in both GSK-HiTDiP and Janssen-BRC studies when not correcting for anxiety.

Table S11. Gene transcriptional network modules were tested for case-control differences in eigengene expression.

Supplementary Figures

Figure S1. Schematic of statistical analysis

Figure S2. Bayesian analysis of genes differentially expressed in patients with MDD compared to healthy controls in both GSK-HiTDiP and Janssen-BRC studies**.**

Figure S3. Statistical correction for effects of body mass index on significance and effect size of case-control differences in the MDD-165 consensus gene set.

Supplementary References

List of MRC Immunopsychiatry Consortium Members

University of Cambridge
Edward Bullmore
Petra E. Vértes
Rudolf Cardinal
Department of Psychiatry, Behavioural and Clinical Neuroscience Institute, University of Cambridge, Cambridge CB2 0SZ

MRC Biostatistics Unit (Cambridge)
Sylvia Richardson
Gwenaél G.R. Leday
MRC Biostatistics Unit, Cambridge Institute of Public Health, Forvie Site, Robinson Way, Cambridge Biomedical Campus, Cambridge CB2 0SR

University of Edinburgh
Tom Freeman
David Hume
Tim Regan
Zhaozong Wu
System Immunology Group, Division of Genetics and Genomics, The Roslin Institute and Royal (Dick) School of Veterinary Studies, University of Edinburgh, Easter Bush, Midlothian EH25 9RG

King's College London
Carmine Pariante
Annamaria Cattaneo
Patricia Zuszain
Alessandra Borsini
Psychiatry and Immunology Lab & Perinatal Psychiatry, Institute of Psychiatry, Psychology and Neuroscience, King's College London G.32.01, The Maurice Wohl Clinical Neuroscience Institute, Cutcombe Road, London SE5 8AF

Robert Stewart
David Chandran
Institute of Psychiatry, Psychology and Neuroscience, King's College London, The Maurice Wohl Clinical Neuroscience Institute, Cutcombe Road, London SE5 9RT

University College London
Livia Carvalho
Joshua Bell
Luis Henrique Souza-Teodoro
Department of Epidemiology and Public Health, UCL, 1-19 Torrington Place, London WC1E 7HB

University of Southampton
Hugh Perry
Centre for Biological Sciences, University of Southampton, Mail Point 840 LD80B, South Lab and Path Block, Southampton General Hospital, SO16 6YD

University of Sussex
Neil Harrison
Brighton & Sussex Medical School, University of Sussex, Brighton BN1 9RR, United Kingdom

Janssen
Wayne Drevets
Gayle M Wittenberg
Yu Sun
Janssen Research & Development, 1125 Trenton-Harbourton Road, Titusville, NJ 08560

Declan Jones
J&J Innovation Centre, One Chapel Place London W1G 0BG

GlaxoSmithKline
Edward Bullmore
Robert B Henderson
ImmunoInflammation Therapeutic Area Unit, GSK R&D, Gunnels Wood Road, Stevenage SG1 2NY

Shahid Khan
Annie Stylianou
GSK R&D, Gunnels Wood Road, Stevenage SG1 2NY

Supplementary Text

Samples

We analysed data from two case-control studies of depression: the GSK-HiTDiP study and the Janssen-BRC study. Other aspects of these studies have been previously reported (1-5) but the microarray data on peripheral gene expression has not been previously reported for the HiTDIP study.

GSK-HiTDiP: This study was designed primarily as a case-control study to identify genetic sequence (DNA) variations associated with a diagnosis of major depressive disorder (MDD). In total, N=1022 Caucasian patients with recurrent MDD were recruited at the Max-Planck Institute of Psychiatry in Munich, Germany, and at two other hospitals in the Munich area (BKH Augsburg and Klinikum Ingolstadt). Patients were evaluated using the semi-structured Schedule for Clinical Assessment in Neuropsychiatry (SCAN; (6)), administered by trained staff. Patients were included if they had a diagnosis of recurrent MDD, i.e., at least two episodes of depression satisfying DSM-IV or ICD10 criteria. Exclusion criteria included: i) experience of mood-incongruent psychotic symptoms or lifetime history of schizophrenia, schizoaffective disorder or other axis 1 disorders; ii) lifetime history of intravenous drug use or diagnosis of drug dependency; iii) lifetime history of depression secondary to alcohol, substance abuse, medical disorders or use of prescribed medication; iv) lifetime history of obsessive compulsive disorder (OCD) or post-traumatic stress disorder (PTSD). Note that history of anxiety symptoms or disorders (apart from OCD and PTSD) was not an exclusion criterion.

The control group comprised 968 Caucasian healthy controls recruited from a Munich-based community sample at the Max-Planck Institute of Psychiatry. All participants were screened for schizophrenia, anxiety and mood disorders using the Composite International Diagnostic Screener (7) and were included if there was no evidence for these disorders.

Micro-array data were available from whole blood on a subset of this sample, comprising patients with MDD (N=128, 64 with generalised anxiety disorder, diagnosed by the MINI questionnaire, and 64 without anxiety disorder) and healthy controls (N=64). RNA was isolated from all samples using the standard PAXgene protocol on the Qiagen Biorobot 8000. All samples gave good quality RNA, as assessed by Agilent Bioanalyser. The yield range was 0.86-15.05ug with an average of 6.25ug. Samples were then randomised into batches, with each batch containing a representative number of controls, depression with anxiety and depression without anxiety, and the same ratio of females to males (3:1). 50ng

of RNA from each sample was converted to a biotin labeled cDNA probe using NuGEN SPIA amplification. The probes were then hybridized to Affymetrix U133_Plus2.0 Genechips.

All participants provided informed consent in writing. The study was approved by an independent ethics review board.

Janssen-BRC: This study was designed to study molecular profiles (gene expression, proteomics and metabolomics) of depressed patients in conjunction with data being captured as part of the BRC International Database to seek the best biomarker profiles for depression including personal and medical history, cognition, and EEG. One hundred (100) participants (66% females) with MDD were recruited from Sydney, New South Wales and Adelaide, South Australia. Participants were referred from general practitioners and psychiatrists. In addition to the 100 clinical subjects, 100 healthy controls matched by age, gender, education, and recruitment site were included. Patients were required to be drug naïve or exceed a compound-specific washout period based on time required for the amount of drug in the patient's system to be less than 3% of the minimum daily therapeutic dose. Depressed patients had a HAM-D17 total score >= 18. Patients with a history of substance abuse in the last year, or with a history of a mental disorder not related to depression were excluded. Anxiety symptoms were reported on the MINI, and symptoms of melancholia were reported by the CORE score (8).

Gene expression was profiled on RNA isolated from whole blood using the Ovation™ Whole Blood Solution. Amplified and labeled RNA were hybridized to the Affymetrix Human Genome U133 Plus 2.0 Array interrogating over 47,000 transcripts. Samples were stored at BRC at -20°C prior to shipping for molecular profiling. Micro-array data were included for patients whose samples were stored for less than 1 year. This comprised patients with MDD (N=94, 40 with generalised anxiety disorder indicated on the MINI) and healthy controls (N=100). All participants provided informed consent in writing. The study was approved by an independent ethics review board.

Patient demographics for patients whose expression data is included in each study are shown in **Supplementary Table S2**.

Microarray data

Whole blood samples from the GSK-HiTDIP and from the Janssen-BRC studies were both analysed using the Affymetrix Human Genome U133 Plus 2.0 Array. Affymetrix CEL files were read and RNA normalized using the R package *affy* (version 1.46.1; (9)). Data were quality controlled using the R package *arrayQualityMetrics* (10) resulting in the exclusion of

22 GSK-HiTDIP samples that failed one or more of the outlier tests. At this stage, the micro-array data consisted of 54,675 probes measured on 113 cases versus 57 controls for GSK-HiTDIP, and on 94 cases versus 100 controls for Janssen-BRC. These matching samples were quantile normalized using the R package *limma* (11). Probes were annotated with the Bioconductor Annotation package hgu133plus2.db (version 3.1.3) and 13,124 unannotated probes were removed. From the remaining 41,551 annotated probes, we selected a unique and optimal probeset per gene using the scoring method (12) implemented in the R package *jetset* (version 3.1.3). This resulted in identification of 18,949 probes from the GSK-HiTDiP study and 18,889 probes from the Janssen-BRC study, which were used for the differential expression analysis in each dataset. For all further analysis we used the data from 18,863 unique probes measured in both studies.

Differential gene expression in each case-control study separately

We first conducted differential expression analysis in each of the two studies, separately; see **Supplementary Figure S1** for schematic overview of data analysis strategy. Each list of *P*-values was thresholded to control the false discovery rate (FDR) at 10%. In doing so we found 133 genes differentially expressed in the GSK-HiTDiP study and 12 genes in the Janssen-BRC study, with no overlapping genes between the two (**Supplementary Tables S1 and S2**). We here remark that the significance level is 10% for each study, but that the significance level for discoveries lying within the intersection between the two thresholded lists of P-values is controlled at a level much lower than 10%. Therefore, to improve the power of our statistical analysis we employed the Bayesian approach of Blangiardo & Richardson (13) that allows the determination of a more lenient *P*-value threshold to yield higher specificity while maintaining good sensitivity.

Combining P-values for differential gene expression from two studies

To identify MDD-related genes that replicate in both GSK-HiTDiP and Janssen-BRC datasets, we statistically assessed the intersection between the probability measures (*P*-values) provided by the differential expression analyses of the two studies. We remark that to do this, it is tempting to use a pre-specified threshold q on the *P*-values in each experiment and carry out a chi-square test of independence between the two sets of significant genes to evaluate whether the degree of overlap is greater than expected by chance. However, this approach relies heavily on the arbitrary choice of q and ignores the levels of evidence for differential expression represented by the *P*-values. For these reasons, we instead adopted the Bayesian method of Blangiardo & Richardson (13), implemented in the R package *sdef* (14). Briefly, this means mapping the *P*-value threshold

q to a ratio $R(q)$ which is proportional to the number of genes differentially expressed in both studies divided by the number expected by chance. By tuning *q* threshold selection to optimise the ratio between observed and expected numbers of genes differentially expressed in both studies, this method confers greater power than more conventional approaches such as simply identifying the genes in common between two lists each thresholded conservatively to control type 1 error. We chose to use the threshold q_2, which represents the largest (most lenient) threshold for which there are at least twice as many significant case-control differences in common between the two studies as expected by chance (**Supplementary Figure S2**). To cross-validate the results of this analysis we also used Fisher's chi square test to combine *P*-values from the GSK-HiTDIP and Janssen-BRC datasets.

The method identifies the *P*-value threshold associated with optimal value of the ratio between the number of significant case-control differences observed at the same genes in both studies *versus* the number of coincidental differences expected by chance. More formally, assuming a fixed (common) *P*-value threshold $0 < q < 1$ for both lists of p *P*-values (corresponding to p genes or probes), we are interested in the following ratio:

$$T(q) = \frac{O_{HB}(q)}{p^{-1} O_B(q) O_H(q)}, \qquad (1)$$

where $O_H(q)$ and $O_B(q)$ are the number of differentially expressed genes in the GSK-HiTDIP and Janssen-BRC data sets, respectively, and $O_{HB}(q)$ is the number of differentially expressed genes in common. $T(q)$ represents the ratio between the observed and expected number of genes in common between the two experiments. Note that marginal frequencies are assumed fixed given *q*. Ideally, the threshold *q* is chosen so as to maximize $T(q)$. However, $T(q)$ may have high variance, in particular when q is small and only a handful of genes are differentially expressed in each experiment. To improve inference about $T(q)$ and the determination of an appropriate threshold, we used a Bayesian approach (13) that fully accounts for the uncertainty in the marginal frequencies. The model assumes that the data O (observed frequencies) arise from a multinomial distribution:

$$Multi(O \mid q, p) \; \mu \; q_1(q)^{O_{HB}(q)} q_2(q)^{O_H(q) - O_{HB}(q)} q_3(q)^{O_B(q) - O_{HB}(q)} q_4(q)^{p - O_H(q) - O_B(q) + O_{HB}(q)} \qquad (2)$$

with a conjugate Dirichlet prior placed on the vector of parameters $\theta(q) \sim Dir(\alpha,\alpha,\alpha,\alpha)$, where *a* = 0.5. The value *a* = 0.5 reflects a high prior variance on frequencies and, hence, will have a relatively small influence on inference. Conjugacy facilitates efficient sampling from the joint posterior distribution. Our interest is in

$$R(q) = \frac{\theta_1(q)}{(\theta_1(q)+\theta_2(q))(\theta_1(q)+\theta_3(q))}, \quad (3)$$

which represents the ratio of the number of differentially expressed genes in common for both experiments divided by the number of genes in common that are expected under the null hypothesis of a multinomial distribution. By sampling from the posterior distribution it is possible to obtain standard summary statistics of the distribution of $R(q)$, including mean, median and credible intervals (CIs). These can then be used to determine an appropriate threshold q. Following Blangiardo and Richardson (13) we used

$$q_2 = \arg\max\{Median(R(q) \mid O, p) \geq 2 : 1 \notin CI_{95}\}, \quad (4)$$

to threshold P-values from the GSK-HiTDIP and Janssen-BRC datasets. q_2 represents the largest threshold for which $Median(R(q) \mid O, p) \geq 2$, i.e., there are at least twice as many case-control differences in common between the two studies as expected by chance, and the 95% credible interval CI_{95} does not include 1 (which would indicate that the observed number of common differences was the same as expected by chance). In simulations, Blangiardo and Richardson (13) demonstrated that thresholding P-value lists based on q_2 yields high specificity while maintaining good sensitivity.

Note that this Bayesian method of threshold setting does not exercise such strong type 1 error (false positive) control as the more established false discovery rate procedures for genome-wide frequentist testing of each gene separately.

To cross-validate the results of this analysis we also used Fisher's statistic, F, to combine *P*-values from the GSK-HiTDIP and Janssen-BRC datasets:

$$F = -2\sum_{s=1}^{S} \log(p_{is}) \sim \chi^2_{2S} \quad (5)$$

where S = 2 denotes the number of experiments and p_{is} denotes the *P*-value for the case-control difference of the i-th gene in the s-th study. For each gene, Fisher's statistic is distributed as chi-square on 2S degrees of freedom and the corresponding list of *P*-values was thresholded by FDR = 10% to define a set of genes significantly expressed in common between both studies, controlling for multiple comparisons (15).

Weighted Gene Correlation Network Analysis (WGCNA; 14, 15)

From the WGCNA software package in R (16), we used the "signed" network option, with the minimum module size set to 50 and options softPower=6 and deepSplit=2. This yielded a normative gene co-expression network with 17 modules, which was visualised using *BioLayout Express3D* software (17, 18). For clarity, we only included links with positive or negative correlation strength greater than 0.7 and nodes connected to the largest component of the graph.

Effects of controlling for comorbid anxiety

In this work, gene expression was primarily adjusted for batch, age, gender and anxiety. When differential expression (within each study) was estimated by a model that did not code for presence or absence of comorbid anxiety, we found 24 differentially expressed genes in the GSK-HiTDIP study and 29 in the Janssen-BRC study, both at 10% FDR. The Bayesian analysis, combining *P*-values from both studies, reported 24 genes that were differentially expressed (without controlling for anxiety) in both studies, of which 21 were fold-change concordant (**Supplementary Table S9**). Although the set of replicable and concordant differentially expressed genes was reduced by not statistically controlling for comorbid anxiety, we still observe some overlap with our previous results and the NESDA study. Among the 21 concordant genes, 15 belong to the list of 165 genes identified by the primary analysis (controlling for anxiety): *MMP8, HP, NRG1, RNASE1, SLPI, CAMP, S100A12, PGLYRP1* and *MGST1*. Two genes (*PGLYRP1* and *NFATC2*) were also differentially expressed in the NESDA study at FDR = 5%. When the FDR threshold for the NESDA study was relaxed to 10% and 20%, 5 and 11 of the 20 genes differentially expressed in both GSK-HiTDiP and Janssen-BRC studies (respectively) were also differentially expressed in the NESDA study (**Supplementary Table S9**). By comparison, we expect to find 0.26, 1.11 and 4.05 genes in common by chance with the NESDA study at FDR thresholds of 5%, 10% and 20%, respectively.

Table S1. Summary of case-control studies of whole genome expression in peripheral blood cells from patients with major depressive disorder (MDD) compared to healthy controls.

Study	Disorder	RNA source	RNA analysis
**GSK-HiTDIP**	Control (n=57)<br>MDD (n=113)	Whole blood	Affymetrix U133 Plus 2.0
**Janssen-BRC**	Control (n=100)<br>MDD (n=94)	Whole blood	Affymetrix U133 Plus 2.0
**Jansen et al. (19)**	Control (n=331)<br>MDD (n=882)	Whole blood	Affymetrix U219
**Mostafavi et al (20)**	Control (n=459)<br>MDD (n=463)	Whole blood	RNA-seq
**Spijker et al. (21)**	Control (n=21)<br>MDD (n=21)	Whole blood;<br>*ex vivo* LPS stimulation	Agilent
**Savitz et al. (22)**	Control (n=24)<br>MDD (n=29)	PBMC	Illumina Human HT-12 v4
**Segman et al (23)**	Control (n=10)<br>Postpartum Depression (n=9)	PBMC	Affymetrix Human Exon 1.0
**Yi et al., 2012 (24)**	Control (n=8)<br>MDD (n=8)	Lymphocytes	Affymetrix U133 Plus 2.0
**Glahn et al (25)**	215 cases	Lymphocytes	Illumina Sentrix Human Whole Genome (WG-6) Series 1 BeadChips

Table S2. Demographic and clinical sample characteristics for both GSK-HiTDiP and Janssen-BRC case-control studies.

	Janssen-BRC			GSK-HiTDIP		
	Control (N=100)	Depressed (N=94)	P-value	Control (N=57)	Depressed (N=113)	P-value
**Female N (%)**	61 (61.0%)	58 (61.7%)	0.9902	16 (28%)	25 (22%)	0.39
**Age Mean (SD)**	38 (15)	39 (14)	0.65	52 (11)	52 (12)	0.96
**HAMD-17**	0.3 (0.7)	22 (4)	**<0.0001**	-	-	
**Anxiety N (%)**	0 (0%)	40 (42.5%)	**<0.0001**	0 (0%)	57 (50%)	**<0.0001**
**Melancholic N (%)**	0 (0%) n=97	26 (27.7%) n=90	**<0.0001**	-	-	
**BMI Mean (SD;N)**	25.5 (4.9) n=80	25.6 (4.5) n=74	0.96	-	-	
**Substance Abuse (%)**	2(7.1%) n=73	28(31.8%) n=88	**<0.0001**			

Note: Only limited socio-demographic and clinical data were available on these samples. In particular, for the GSK-HiTDiP study, which was designed primarily as a genetic association study, clinical phenotyping data were not available on participants. Neither study collected data on drug or alcohol consumption, ethnicity, or exposure to immuno-modulatory medication. In BRC, CORE score, BMI and substance abuse were missing for some subjects, as indicated above. Melancholia was defined as CORE >=8. P-values were chi-squared for gender, anxiety, melancholia, and substance abuse, two-tailed t-test for age, HAMD-17, and BMI.

Table S3. List of differentially expressed genes in the GSK-HiTDIP data set. Genes are ranked according to their FDR < 0.1, estimated parametrically by the limma software package. *P*-values and FDR are also reported by permutation tests. FC = fold change of gene expression in MDD compared to healthy controls; FC CI = FC 95% confidence interval.

Rank	Probe ID	Symbol	FC	FC_CI	Limma P	Limma FDR	Limma < 5% FDR	Perm. P	Perm. FDR
**1**	204924_at	TLR2	1.384	[1.225,1.563]	4.14E-07	4.23E-03	1	0	0
**2**	226733_at	PFKFB2	1.588	[1.334,1.889]	4.46E-07	4.23E-03	1	0	0
**3**	219870_at	ATF7IP2	0.754	[0.673,0.844]	2.01E-06	1.27E-02	1	0	0
**4**	200663_at	CD63	1.235	[1.131,1.349]	5.05E-06	1.94E-02	1	0	0
**5**	223832_s_at	CAPNS2	1.471	[1.252,1.73]	5.13E-06	1.94E-02	1	0	0
**6**	218865_at	MARC1	1.553	[1.289,1.871]	6.18E-06	1.95E-02	1	0	0
**7**	209409_at	GRB10	1.519	[1.268,1.819]	9.15E-06	2.24E-02	1	0	0
**8**	213201_s_at	TNNT1	1.809	[1.4,2.337]	9.46E-06	2.24E-02	1	0	0
**9**	205863_at	S100A12	1.532	[1.271,1.848]	1.26E-05	2.66E-02	1	0	0
**10**	215049_x_at	CD163	1.421	[1.216,1.66]	1.47E-05	2.67E-02	1	0	0
**11**	202207_at	ARL4C	0.790	[0.712,0.877]	1.55E-05	2.67E-02	1	2.00E-04	4.92E-02
**12**	224818_at	SORT1	1.343	[1.176,1.533]	1.97E-05	2.95E-02	1	0	0
**13**	201887_at	IL13RA1	1.291	[1.151,1.448]	2.06E-05	2.95E-02	1	0	0
**14**	207809_s_at	ATP6AP1	1.269	[1.138,1.414]	2.56E-05	2.95E-02	1	0	0
**15**	223393_s_at	TSHZ3	1.463	[1.23,1.74]	2.60E-05	2.95E-02	1	1.00E-04	3.27E-02
**16**	224466_s_at	MAFG	1.260	[1.133,1.4]	2.67E-05	2.95E-02	1	0	0
**17**	228345_at	CHIC1	0.714	[0.611,0.833]	2.91E-05	2.95E-02	1	0	0
**18**	1554503_a_at	OSCAR	1.414	[1.205,1.659]	3.11E-05	2.95E-02	1	1.00E-04	3.27E-02
**19**	220570_at	RETN	1.307	[1.155,1.479]	3.17E-05	2.95E-02	1	0	0
**20**	207075_at	NLRP3	1.340	[1.171,1.535]	3.25E-05	2.95E-02	1	0	0
**21**	209262_s_at	NR2F6	1.387	[1.192,1.615]	3.41E-05	2.95E-02	1	0	0
**22**	207651_at	GPR171	0.749	[0.655,0.856]	3.42E-05	2.95E-02	1	0	0
**23**	207236_at	ZNF345	0.778	[0.692,0.875]	3.76E-05	3.07E-02	1	0	0
**24**	203795_s_at	BCL7A	0.698	[0.59,0.826]	3.89E-05	3.07E-02	1	0	0
**25**	204039_at	CEBPA	1.324	[1.16,1.512]	4.51E-05	3.42E-02	1	1.00E-04	3.27E-02
**26**	203006_at	INPP5A	1.266	[1.133,1.416]	4.81E-05	3.50E-02	1	0	0
**27**	235369_at	C14orf28	0.653	[0.533,0.8]	5.38E-05	3.64E-02	1	1.00E-04	3.27E-02
**28**	227072_at	RTTN	0.732	[0.631,0.849]	5.45E-05	3.64E-02	1	0	0
**29**	204140_at	TPST1	1.580	[1.27,1.966]	5.63E-05	3.64E-02	1	0	0
**30**	227672_at	MROH6	1.285	[1.14,1.449]	5.88E-05	3.64E-02	1	0	0
**31**	241353_s_at	LOC100507507	1.291	[1.142,1.459]	5.95E-05	3.64E-02	1	2.00E-04	4.92E-02
**32**	237291_at	PRORSD1P	0.728	[0.625,0.848]	6.15E-05	3.64E-02	1	1.00E-04	3.27E-02
**33**	218454_at	PLBD1	1.273	[1.133,1.431]	7.00E-05	4.02E-02	1	0	0
**34**	206666_at	GZMK	0.672	[0.553,0.816]	7.93E-05	4.42E-02	1	0	0
**35**	228253_at	LOXL3	1.258	[1.124,1.409]	9.22E-05	4.99E-02	1	3.00E-04	6.39E-02
**36**	212705_x_at	PNPLA2	1.249	[1.118,1.396]	1.15E-04	6.06E-02	0	1.00E-04	3.27E-02
**37**	218910_at	ANO10	1.327	[1.152,1.53]	1.20E-04	6.15E-02	0	0	0
**38**	205553_s_at	CSRP3	1.251	[1.118,1.401]	1.32E-04	6.58E-02	0	0	0
**39**	206697_s_at	HP	1.779	[1.329,2.381]	1.39E-04	6.66E-02	0	2.00E-04	4.92E-02
**40**	223703_at	C10orf11	1.358	[1.163,1.587]	1.43E-04	6.66E-02	0	0	0
**41**	228499_at	PFKFB4	1.358	[1.163,1.586]	1.44E-04	6.66E-02	0	1.00E-04	3.27E-02
**42**	205349_at	GNA15	1.331	[1.151,1.54]	1.52E-04	6.79E-02	0	1.00E-04	3.27E-02
**43**	226682_at	RORA	0.610	[0.474,0.785]	1.54E-04	6.79E-02	0	1.00E-04	3.27E-02
**44**	206155_at	ABCC2	1.299	[1.136,1.485]	1.61E-04	6.94E-02	0	2.00E-04	4.92E-02
**45**	201007_at	HADHB	1.130	[1.061,1.203]	1.68E-04	6.97E-02	0	1.00E-04	3.27E-02

Rank	Probe ID	Symbol	FC	FC_CI	Limma P	Limma FDR	Limma < 5% FDR	Perm. P	Perm. FDR
**46**	218764_at	PRKCH	0.813	[0.73,0.904]	1.71E-04	6.97E-02	0	1.00E-04	3.27E-02
**47**	238025_at	MLKL	1.229	[1.105,1.366]	1.73E-04	6.97E-02	0	1.00E-04	3.27E-02
**48**	32541_at	PPP3CC	0.837	[0.764,0.918]	1.82E-04	7.06E-02	0	1.00E-04	3.27E-02
**49**	230695_s_at	RSPH9	1.377	[1.168,1.624]	1.83E-04	7.06E-02	0	1.00E-04	3.27E-02
**50**	211121_s_at	DOK1	1.226	[1.103,1.363]	1.90E-04	7.20E-02	0	1.00E-04	3.27E-02
**51**	212658_at	LHFPL2	1.323	[1.144,1.529]	1.97E-04	7.30E-02	0	3.00E-04	6.39E-02
**52**	201968_s_at	PGM1	1.192	[1.088,1.306]	2.02E-04	7.30E-02	0	0	0
**53**	224576_at	ERGIC1	1.158	[1.073,1.25]	2.05E-04	7.30E-02	0	2.00E-04	4.92E-02
**54**	1554149_at	CLDND1	0.785	[0.692,0.891]	2.14E-04	7.30E-02	0	0	0
**55**	206310_at	SPINK2	0.760	[0.658,0.877]	2.19E-04	7.30E-02	0	2.00E-04	4.92E-02
**56**	240313_at	DMRTB1	1.164	[1.075,1.261]	2.32E-04	7.30E-02	0	0	0
**57**	229295_at	IL17RA	1.204	[1.092,1.327]	2.33E-04	7.30E-02	0	1.00E-04	3.27E-02
**58**	204440_at	CD83	0.746	[0.64,0.871]	2.42E-04	7.30E-02	0	1.00E-04	3.27E-02
**59**	209467_s_at	MKNK1	1.248	[1.111,1.403]	2.43E-04	7.30E-02	0	1.00E-04	3.27E-02
**60**	208130_s_at	TBXAS1	1.276	[1.122,1.45]	2.46E-04	7.30E-02	0	2.00E-04	4.92E-02
**61**	219938_s_at	PSTPIP2	1.228	[1.102,1.368]	2.50E-04	7.30E-02	0	0	0
**62**	203437_at	TMEM11	1.148	[1.067,1.234]	2.54E-04	7.30E-02	0	1.00E-04	3.27E-02
**63**	1568898_at	LOC1019 28140	0.833	[0.756,0.917]	2.55E-04	7.30E-02	0	3.00E-04	6.39E-02
**64**	207205_at	CEACAM 4	1.468	[1.198,1.798]	2.60E-04	7.30E-02	0	3.00E-04	6.39E-02
**65**	209114_at	TSPAN1	1.176	[1.079,1.281]	2.65E-04	7.30E-02	0	3.00E-04	6.39E-02
**66**	203535_at	S100A9	1.208	[1.093,1.335]	2.68E-04	7.30E-02	0	2.00E-04	4.92E-02
**67**	210279_at	GPR18	0.798	[0.707,0.899]	2.73E-04	7.30E-02	0	6.00E-04	8.68E-02
**68**	236172_at	LTB4R	1.383	[1.164,1.642]	2.73E-04	7.30E-02	0	6.00E-04	8.68E-02
**69**	219259_at	SEMA4A	1.323	[1.14,1.535]	2.75E-04	7.30E-02	0	6.00E-04	8.68E-02
**70**	232933_at	KIAA1656	1.230	[1.102,1.373]	2.75E-04	7.30E-02	0	2.00E-04	4.92E-02
**71**	1553952_at	ZDHHC1 9	1.264	[1.116,1.432]	2.76E-04	7.30E-02	0	2.00E-04	4.92E-02
**72**	205708_s_at	TRPM2	1.245	[1.108,1.399]	2.83E-04	7.30E-02	0	6.00E-04	8.68E-02
**73**	214470_at	KLRB1	0.731	[0.618,0.864]	2.85E-04	7.30E-02	0	2.00E-04	4.92E-02
**74**	215967_s_at	LY9	0.800	[0.71,0.901]	2.85E-04	7.30E-02	0	8.00E-04	9.78E-02
**75**	228442_at	NFATC2	0.801	[0.712,0.902]	2.96E-04	7.48E-02	0	2.00E-04	4.92E-02
**76**	203754_s_at	BRF1	1.190	[1.084,1.307]	3.18E-04	7.79E-02	0	5.00E-04	8.31E-02
**77**	213198_at	ACVR1B	1.284	[1.123,1.468]	3.20E-04	7.79E-02	0	2.00E-04	4.92E-02
**78**	224918_x_at	MGST1	1.323	[1.138,1.538]	3.28E-04	7.79E-02	0	2.00E-04	4.92E-02
**79**	221483_s_at	ARPP19	0.855	[0.786,0.93]	3.28E-04	7.79E-02	0	0	0
**80**	219177_at	BRIX1	0.792	[0.698,0.898]	3.29E-04	7.79E-02	0	1.00E-04	3.27E-02
**81**	228920_at	ZNF260	0.748	[0.64,0.875]	3.47E-04	8.10E-02	0	6.00E-04	8.68E-02
**82**	213216_at	OTUD3	0.761	[0.656,0.882]	3.52E-04	8.10E-02	0	0	0
**83**	200919_at	PHC2	1.325	[1.137,1.543]	3.59E-04	8.10E-02	0	2.00E-04	4.92E-02
**84**	200661_at	CTSA	1.257	[1.111,1.424]	3.59E-04	8.10E-02	0	2.00E-04	4.92E-02
**85**	206111_at	RNASE2	1.564	[1.227,1.993]	3.67E-04	8.10E-02	0	4.00E-04	7.43E-02
**86**	236407_at	KCNE1	1.531	[1.215,1.93]	3.68E-04	8.10E-02	0	9.00E-04	9.97E-02
**87**	209694_at	PTS	0.727	[0.612,0.865]	3.72E-04	8.10E-02	0	4.00E-04	7.43E-02
**88**	239237_at	TRG-AS1	0.762	[0.657,0.883]	3.77E-04	8.11E-02	0	8.00E-04	9.78E-02
**89**	1560625_s_a t	CATIP-AS1	1.380	[1.158,1.645]	3.81E-04	8.11E-02	0	6.00E-04	8.68E-02
**90**	217815_at	SUPT16H	0.890	[0.836,0.949]	4.03E-04	8.48E-02	0	1.00E-04	3.27E-02
**91**	220507_s_at	UPB1	1.333	[1.139,1.56]	4.07E-04	8.48E-02	0	5.00E-04	8.31E-02
**92**	205633_s_at	ALAS1	1.248	[1.105,1.408]	4.13E-04	8.48E-02	0	3.00E-04	6.39E-02
**93**	208487_at	LMX1B	1.182	[1.079,1.296]	4.16E-04	8.48E-02	0	6.00E-04	8.68E-02
**94**	212531_at	LCN2	2.118	[1.403,3.198]	4.21E-04	8.49E-02	0	3.00E-04	6.39E-02
**95**	223095_at	MARVEL D1	1.283	[1.119,1.47]	4.27E-04	8.52E-02	0	3.00E-04	6.39E-02

Rank	Probe ID	Symbol	FC	FC_CI	Limma P	Limma FDR	Limma < 5% FDR	Perm. P	Perm. FDR
**96**	218284_at	SMAD3	0.818	[0.733,0.914]	4.37E-04	8.57E-02	0	1.00E-04	3.27E-02
**97**	200986_at	SERPING1	1.834	[1.313,2.561]	4.39E-04	8.57E-02	0	4.00E-04	7.43E-02
**98**	219172_at	UBTD1	1.227	[1.096,1.374]	4.47E-04	8.64E-02	0	6.00E-04	8.68E-02
**99**	227667_at	CUEDC1	1.229	[1.097,1.378]	4.69E-04	8.98E-02	0	3.00E-04	6.39E-02
**100**	220284_at	DKKL1	1.223	[1.094,1.367]	4.74E-04	8.99E-02	0	5.00E-04	8.31E-02
**101**	225391_at	LOC93622	0.834	[0.755,0.923]	4.91E-04	9.20E-02	0	6.00E-04	8.68E-02
**102**	228176_at	S1PR3	1.483	[1.191,1.847]	4.96E-04	9.21E-02	0	6.00E-04	8.68E-02
**103**	200766_at	CTSD	1.274	[1.114,1.458]	5.02E-04	9.24E-02	0	4.00E-04	7.43E-02
**104**	237350_at	TTC36	1.219	[1.091,1.36]	5.11E-04	9.25E-02	0	4.00E-04	7.43E-02
**105**	226500_at	ZBTB47	1.227	[1.095,1.374]	5.13E-04	9.25E-02	0	4.00E-04	7.43E-02
**106**	206237_s_at	NRG1	1.570	[1.221,2.021]	5.26E-04	9.41E-02	0	8.00E-04	9.78E-02
**107**	231151_at	DLGAP3	1.219	[1.091,1.362]	5.38E-04	9.53E-02	0	3.00E-04	6.39E-02
**108**	219751_at	SETD6	0.808	[0.717,0.91]	5.46E-04	9.59E-02	0	5.00E-04	8.31E-02
**109**	208928_at	POR	1.249	[1.103,1.415]	5.54E-04	9.64E-02	0	8.00E-04	9.78E-02
**110**	226956_at	MTMR3	1.244	[1.1,1.406]	5.77E-04	9.68E-02	0	8.00E-04	9.78E-02
**111**	212252_at	CAMKK2	1.220	[1.091,1.364]	5.80E-04	9.68E-02	0	3.00E-04	6.39E-02
**112**	M33197_3_at	GAPDH	1.141	[1.059,1.229]	5.89E-04	9.68E-02	0	2.00E-04	4.92E-02
**113**	204562_at	IRF4	0.804	[0.711,0.909]	5.91E-04	9.68E-02	0	4.00E-04	7.43E-02
**114**	212614_at	ARID5B	0.756	[0.645,0.885]	5.92E-04	9.68E-02	0	3.00E-04	6.39E-02
**115**	216667_at	ECRP	1.433	[1.17,1.756]	6.01E-04	9.68E-02	0	8.00E-04	9.78E-02
**116**	205645_at	REPS2	1.356	[1.142,1.611]	6.10E-04	9.68E-02	0	8.00E-04	9.78E-02
**117**	225282_at	SMAP2	1.150	[1.063,1.244]	6.10E-04	9.68E-02	0	7.00E-04	9.28E-02
**118**	201118_at	PGD	1.279	[1.113,1.471]	6.19E-04	9.68E-02	0	9.00E-04	9.97E-02
**119**	242794_at	MAML3	1.337	[1.134,1.576]	6.25E-04	9.68E-02	0	6.00E-04	8.68E-02
**120**	225379_at	MAPT	1.208	[1.085,1.344]	6.36E-04	9.68E-02	0	2.00E-04	4.92E-02
**121**	212494_at	TENC1	1.227	[1.093,1.379]	6.37E-04	9.68E-02	0	5.00E-04	8.31E-02
**122**	205819_at	MARCO	1.350	[1.139,1.601]	6.37E-04	9.68E-02	0	5.00E-04	8.31E-02
**123**	230725_at	ANKRD32	1.268	[1.108,1.45]	6.41E-04	9.68E-02	0	5.00E-04	8.31E-02
**124**	209588_at	EPHB2	1.350	[1.138,1.6]	6.44E-04	9.68E-02	0	7.00E-04	9.28E-02
**125**	227119_at	CNOT6L	0.833	[0.751,0.924]	6.46E-04	9.68E-02	0	4.00E-04	7.43E-02
**126**	243690_at	TRIOBP	1.283	[1.114,1.479]	6.47E-04	9.68E-02	0	2.00E-04	4.92E-02
**127**	209263_x_at	TSPAN4	1.238	[1.097,1.397]	6.49E-04	9.68E-02	0	4.00E-04	7.43E-02
**128**	1556209_at	CLEC2B	0.812	[0.721,0.914]	6.59E-04	9.68E-02	0	4.00E-04	7.43E-02
**129**	210152_at	LILRB4	1.253	[1.102,1.424]	6.60E-04	9.68E-02	0	1.20E-03	1.09E-01
**130**	201700_at	CCND3	1.181	[1.074,1.298]	6.64E-04	9.68E-02	0	7.00E-04	9.28E-02
**131**	205513_at	TCN1	1.677	[1.248,2.253]	6.87E-04	9.92E-02	0	9.00E-04	9.97E-02
**132**	1569009_s_at	LOC100132249	1.161	[1.066,1.264]	6.95E-04	9.92E-02	0	4.00E-04	7.43E-02
**133**	203021_at	SLPI	1.586	[1.218,2.064]	6.96E-04	9.92E-02	0	7.00E-04	9.28E-02

Table S4. List of differentially expressed genes in the Janssen-BRC data set. Genes are ranked according to their FDR < 0.1, estimated parametrically by the *limma* software package. *P*-values and FDR are also reported by permutation tests. FC = fold change of gene expression in MDD compared to healthy controls; FC CI = FC 95% confidence interval.

Rank	Probe ID	Symbol	FC	FC CI	Limma P	Limma FDR	< 5% FDR	Perm. P	Perm. FDR
**1**	209045_at	XPNPEP1	0.884	[0.838,0.932]	8.78E-06	8.16E-02	0	0	0
**2**	224318_s_at	KANSL3	0.868	[0.815,0.924]	1.49E-05	8.16E-02	0	0	0
**3**	224414_s_at	CARD6	1.225	[1.117,1.344]	2.39E-05	8.16E-02	0	1.00E-04	7.87E-02
**4**	224471_s_at	BTRC	0.867	[0.812,0.925]	2.44E-05	8.16E-02	0	0	0
**5**	224302_s_at	MRPS36	1.133	[1.069,1.2]	3.13E-05	8.16E-02	0	0	0
**6**	204790_at	SMAD7	0.784	[0.701,0.878]	3.30E-05	8.16E-02	0	1.00E-04	7.87E-02
**7**	217728_at	S100A6	1.164	[1.084,1.249]	3.65E-05	8.16E-02	0	0	0
**8**	225522_at	AAK1	0.857	[0.798,0.921]	3.81E-05	8.16E-02	0	0	0
**9**	231844_at	MGC27345	0.861	[0.802,0.923]	3.89E-05	8.16E-02	0	0	0
**10**	225947_at	MYO19	0.851	[0.789,0.919]	4.86E-05	8.96E-02	0	0	0
**11**	210244_at	CAMP	1.571	[1.266,1.948]	5.30E-05	8.96E-02	0	0	0
**12**	1554582_a_at	METTL20	1.326	[1.159,1.518]	5.69E-05	8.96E-02	0	1.00E-04	7.87E-02

Table S5. List of 165 genes that are significantly differentially expressed (q_2 = 0.02) with consistent sign of fold change in both the GSK-HiTDIP and Janssen-BRC studies. This list was defined using a Bayesian rule for finding the largest *P*-value threshold associated with twice as many differentially expressed genes in both studies as would be expected by random coincidence (implemented in sdef software; Blangiardo et al [14]). Genes are ranked according to the HiTDIP fold-change (FC). The last four columns indicate genes that are "in NESDA", i.e., that were measured by the microarrays used in the NESDA study, and those among them that were replicated in the NESDA study at FDR thresholds of 5% (7 genes), 10% (18 genes) and 20% (45 genes). FC CI = FC 95% confidence interval.

Rank	Symbol	HiTDIP FC	HiTDIP FC CI	HiTDIP P	BRC FC	BRC FC CI	BRC P	In NESDA	NESDA 5%	NESDA 10%	NESDA 20%
**1**	LCN2	2.118	[1.403, 3.198]	4.21E-04	1.605	[1.121, 2.298]	9.99E-03	1	0	0	0
**2**	OLFM4	2.109	[1.115, 3.988]	2.20E-02	1.948	[1.156, 3.283]	1.26E-02	1	0	0	0
**3**	DEFA4	2.102	[1.288, 3.431]	3.16E-03	1.703	[1.112, 2.61]	1.47E-02	1	0	0	0
**4**	MMP8	1.972	[1.33,2.923]	8.23E-04	1.902	[1.286, 2.813]	1.42E-03	1	0	0	0
**5**	CEACAM8	1.916	[1.172, 3.133]	9.79E-03	1.687	[1.129, 2.522]	1.11E-02	1	0	0	0
**6**	BPI	1.796	[1.225, 2.634]	2.91E-03	1.448	[1.073, 1.952]	1.56E-02	1	0	0	0
**7**	RNASE3	1.781	[1.247, 2.543]	1.67E-03	1.773	[1.207, 2.605]	3.69E-03	1	0	0	0
**8**	HP	1.779	[1.329, 2.381]	1.39E-04	1.712	[1.292, 2.268]	2.20E-04	1	0	0	0
**9**	CRISP3	1.662	[1.108, 2.495]	1.44E-02	1.714	[1.199, 2.45]	3.31E-03	1	0	0	0
**10**	CEACAM6	1.631	[1.148, 2.319]	6.65E-03	1.602	[1.121, 2.291]	9.98E-03	1	0	0	0
**11**	TDRD9	1.615	[1.226, 2.128]	7.43E-04	1.329	[1.062, 1.663]	1.30E-02	1	0	0	0
**12**	SLPI	1.586	[1.218, 2.064]	6.96E-04	1.546	[1.194, 2.002]	1.05E-03	1	0	1	1
**13**	NRG1	1.570	[1.221, 2.021]	5.26E-04	1.384	[1.054, 1.817]	1.98E-02	1	0	1	1
**14**	RNASE2	1.564	[1.227, 1.993]	3.67E-04	1.368	[1.109, 1.688]	3.63E-03	1	0	0	0
**15**	S100A12	1.532	[1.271, 1.848]	1.26E-05	1.295	[1.103, 1.522]	1.77E-03	1	0	0	1
**16**	MMP9	1.500	[1.127, 1.998]	5.75E-03	1.343	[1.039, 1.735]	2.44E-02	1	0	1	1
**17**	PGLYRP1	1.466	[1.168, 1.84]	1.09E-03	1.439	[1.174, 1.765]	5.24E-04	1	1	1	1
**18**	RNASE1	1.452	[1.169, 1.804]	8.45E-04	1.523	[1.205, 1.926]	5.01E-04	1	0	0	0
**19**	GPR84	1.435	[1.137, 1.811]	2.53E-03	1.328	[1.086, 1.624]	5.99E-03	1	0	0	0
**20**	ECRP	1.433	[1.17,1.756]	6.01E-04	1.296	[1.066, 1.574]	9.44E-03	0	0	0	0
**21**	CAMP	1.432	[1.13,1.815]	3.18E-03	1.571	[1.266, 1.948]	5.30E-05	1	0	0	1
**22**	MCEMP1	1.355	[1.072, 1.712]	1.14E-02	1.351	[1.114, 1.639]	2.40E-03	0	0	0	0
**23**	ROPN1L	1.334	[1.083, 1.644]	6.94E-03	1.262	[1.048, 1.52]	1.44E-02	1	0	0	0
**24**	CEBPA	1.324	[1.16,1.512]	4.51E-05	1.144	[1.02,1.283]	2.14E-02	1	0	0	0
**25**	MGST1	1.323	[1.138, 1.538]	3.28E-04	1.226	[1.099, 1.368]	3.12E-04	1	0	0	0
**26**	ASGR2	1.322	[1.112, 1.571]	1.69E-03	1.285	[1.089, 1.517]	3.17E-03	1	0	0	0
**27**	ELANE	1.310	[1.052, 1.63]	1.59E-02	1.413	[1.048, 1.905]	2.36E-02	1	0	0	0
**28**	RETN	1.307	[1.155, 1.479]	3.17E-05	1.197	[1.026, 1.396]	2.21E-02	1	0	0	0
**29**	SULT1B1	1.302	[1.052, 1.611]	1.55E-02	1.239	[1.045, 1.469]	1.42E-02	1	0	0	1

**30**	TLR5	1.294	[1.082, 1.547]	5.02E-03	1.197	[1.026, 1.396]	2.21E-02	1	0	0	0
**31**	ATG7	1.292	[1.072, 1.556]	7.36E-03	1.140	[1.03,1.261]	1.14E-02	1	0	1	1
**32**	HK3	1.281	[1.097, 1.496]	1.90E-03	1.162	[1.03,1.311]	1.50E-02	1	0	0	1
**33**	CAPG	1.268	[1.093, 1.473]	1.97E-03	1.179	[1.053, 1.321]	4.57E-03	1	0	0	0
**34**	TP53I3	1.267	[1.092, 1.47]	2.01E-03	1.234	[1.077, 1.412]	2.53E-03	1	0	0	0
**35**	NAIP	1.262	[1.036, 1.537]	2.09E-02	1.211	[1.051, 1.396]	8.25E-03	0	0	0	0
**36**	TSEN34	1.262	[1.095, 1.454]	1.49E-03	1.147	[1.036, 1.27]	8.80E-03	1	0	0	0
**37**	AGTRAP	1.251	[1.093, 1.433]	1.33E-03	1.149	[1.035, 1.275]	9.15E-03	1	0	0	0
**38**	IMPDH1	1.250	[1.072, 1.458]	4.60E-03	1.121	[1.023, 1.228]	1.48E-02	1	0	0	0
**39**	MKNK1	1.248	[1.111, 1.403]	2.43E-04	1.100	[1.013, 1.194]	2.37E-02	1	0	0	0
**40**	CHIT1	1.242	[1.081, 1.426]	2.39E-03	1.268	[1.052, 1.529]	1.29E-02	1	0	0	0
**41**	PYCARD	1.240	[1.073, 1.432]	3.75E-03	1.135	[1.033, 1.247]	8.68E-03	1	0	0	0
**42**	HIST1H2BK	1.238	[1.062, 1.441]	6.45E-03	1.132	[1.021, 1.255]	1.86E-02	1	0	0	0
**43**	PLIN3	1.233	[1.07,1.421]	4.05E-03	1.133	[1.017, 1.262]	2.34E-02	1	0	0	0
**44**	CYSTM1	1.228	[1.028, 1.467]	2.40E-02	1.250	[1.082, 1.443]	2.56E-03	1	0	0	0
**45**	LGALS1	1.226	[1.068, 1.408]	4.06E-03	1.167	[1.05,1.297]	4.26E-03	1	0	0	0
**46**	CPNE2	1.226	[1.046, 1.438]	1.25E-02	1.137	[1.019, 1.268]	2.18E-02	1	0	0	0
**47**	TSPO	1.224	[1.071, 1.399]	3.14E-03	1.153	[1.047, 1.27]	3.92E-03	1	0	0	0
**48**	RAB32	1.218	[1.063, 1.396]	4.84E-03	1.128	[1.021, 1.246]	1.80E-02	1	0	0	0
**49**	LINC01503	1.218	[1.033, 1.436]	1.95E-02	1.235	[1.089, 1.401]	1.15E-03	0	0	0	0
**50**	CTSH	1.215	[1.062, 1.389]	4.76E-03	1.157	[1.046, 1.28]	4.90E-03	1	0	0	0
**51**	CARD6	1.209	[1.033, 1.416]	1.86E-02	1.225	[1.117, 1.344]	2.39E-05	1	0	0	1
**52**	MAP2K6	1.208	[1.047, 1.394]	9.98E-03	1.150	[1.037, 1.274]	8.21E-03	1	0	0	0
**53**	S100A9	1.208	[1.093, 1.335]	2.68E-04	1.133	[1.048, 1.225]	1.87E-03	1	0	0	1
**54**	TFEB	1.203	[1.056, 1.371]	5.66E-03	1.146	[1.031, 1.274]	1.18E-02	1	0	0	0
**55**	SFN	1.202	[1.081, 1.337]	7.88E-04	1.183	[1.067, 1.312]	1.54E-03	1	0	1	1
**56**	PQLC1	1.200	[1.053, 1.368]	6.53E-03	1.148	[1.064, 1.239]	4.22E-04	1	0	0	0
**57**	SV2B	1.198	[1.069, 1.341]	2.00E-03	1.126	[1.031, 1.229]	8.45E-03	1	0	0	0
**58**	TXN	1.196	[1.054, 1.357]	5.71E-03	1.149	[1.027, 1.285]	1.53E-02	1	0	0	0
**59**	CLEC4A	1.191	[1.048, 1.353]	7.71E-03	1.139	[1.04,1.248]	5.29E-03	1	0	0	0
**60**	MAPK14	1.189	[1.054, 1.343]	5.29E-03	1.103	[1.018, 1.197]	1.74E-02	1	1	1	1
**61**	PCOLCE2	1.185	[1.044, 1.346]	8.89E-03	1.209	[1.048, 1.394]	9.39E-03	1	0	0	0
**62**	TMEM120A	1.184	[1.029, 1.364]	1.90E-02	1.147	[1.028, 1.28]	1.45E-02	1	0	0	0
**63**	PSMB3	1.178	[1.06,1.311]	2.68E-03	1.110	[1.032, 1.194]	5.32E-03	1	0	0	0
**64**	PELO	1.178	[1.026, 1.353]	2.03E-02	1.168	[1.051, 1.298]	4.10E-03	1	0	0	0
**65**	ADM	1.177	[1.024, 1.354]	2.20E-02	1.254	[1.085, 1.45]	2.41E-03	1	0	0	0
**66**	ZDHHC12	1.174	[1.043, 1.323]	8.39E-03	1.127	[1.032, 1.231]	7.89E-03	1	0	0	0

**67**	BRI3	1.170	[1.032, 1.326]	1.45E-02	1.118	[1.029, 1.215]	8.50E-03	1	0	0	0
**68**	MXD3	1.163	[1.041, 1.299]	7.82E-03	1.109	[1.019, 1.207]	1.70E-02	1	0	0	0
**69**	RTFDC1	1.162	[1.056, 1.28]	2.28E-03	1.078	[1.013, 1.148]	1.79E-02	0	0	0	0
**70**	KLHL4	1.161	[1.054, 1.279]	2.64E-03	1.113	[1.038, 1.194]	2.97E-03	1	0	0	0
**71**	GDE1	1.160	[1.029, 1.307]	1.52E-02	1.119	[1.032, 1.213]	6.49E-03	1	0	0	1
**72**	S100A6	1.158	[1.034, 1.297]	1.14E-02	1.164	[1.084, 1.249]	3.65E-05	1	0	0	0
**73**	CKLF	1.156	[1.051, 1.271]	3.06E-03	1.158	[1.046, 1.281]	4.87E-03	1	0	0	0
**74**	PLEKHN1	1.145	[1.027, 1.277]	1.52E-02	1.103	[1.013, 1.201]	2.38E-02	1	0	0	1
**75**	NAA38	1.141	[1.025, 1.27]	1.61E-02	1.103	[1.022, 1.191]	1.23E-02	1	0	0	1
**76**	GSTO1	1.140	[1.032, 1.26]	1.04E-02	1.109	[1.035, 1.189]	3.65E-03	0	0	0	0
**77**	TWF2	1.138	[1.026, 1.262]	1.44E-02	1.101	[1.022, 1.186]	1.19E-02	1	0	0	0
**78**	HIST1H1B	1.128	[1.022, 1.245]	1.67E-02	1.090	[1.013, 1.172]	2.09E-02	1	0	0	0
**79**	PREX2	1.128	[1.022, 1.245]	1.69E-02	1.089	[1.016, 1.167]	1.57E-02	1	0	0	0
**80**	S100A8	1.127	[1.022, 1.242]	1.69E-02	1.080	[1.011, 1.154]	2.30E-02	1	0	1	1
**81**	TBCC	1.126	[1.026, 1.236]	1.28E-02	1.094	[1.023, 1.169]	8.61E-03	1	0	0	0
**82**	LINC00330	1.125	[1.029, 1.231]	1.03E-02	1.158	[1.032, 1.299]	1.27E-02	0	0	0	0
**83**	MCU	1.125	[1.028, 1.231]	1.10E-02	1.076	[1.01,1 .146]	2.37E-02	1	0	1	1
**84**	LOC101928 443	1.122	[1.016, 1.239]	2.32E-02	1.097	[1.013, 1.188]	2.33E-02	0	0	0	0
**85**	GALE	1.121	[1.018, 1.235]	2.07E-02	1.105	[1.03,1 .185]	5.49E-03	1	0	0	0
**86**	IL17RC	1.119	[1.019, 1.229]	1.90E-02	1.100	[1.013, 1.195]	2.31E-02	1	0	0	0
**87**	PARD6A	1.111	[1.015, 1.216]	2.31E-02	1.097	[1.018, 1.183]	1.57E-02	1	0	0	0
**88**	GNG5	1.110	[1.039, 1.187]	2.28E-03	1.049	[1.009, 1.092]	1.72E-02	1	0	0	0
**89**	DCC	1.085	[1.011, 1.164]	2.31E-02	1.137	[1.057, 1.223]	6.55E-04	1	0	0	0
**90**	GABARAP	1.081	[1.03,1. 134]	1.68E-03	1.045	[1.012, 1.08]	7.91E-03	1	0	0	0
**91**	ZBTB4	0.928	[0.87,0. 99]	2.39E-02	0.931	[0.886, 0.977]	4.03E-03	1	0	1	1
**92**	OSBP	0.915	[0.854, 0.98]	1.19E-02	0.943	[0.902, 0.986]	9.59E-03	1	0	0	0
**93**	QRICH1	0.913	[0.851, 0.978]	9.91E-03	0.925	[0.878, 0.974]	3.51E-03	1	0	0	0
**94**	P2RY8	0.909	[0.853, 0.969]	3.87E-03	0.924	[0.886, 0.963]	2.67E-04	1	0	0	0
**95**	ASXL1	0.906	[0.836, 0.982]	1.65E-02	0.922	[0.868, 0.979]	8.55E-03	1	0	0	0
**96**	REPS1	0.902	[0.828, 0.982]	1.75E-02	0.905	[0.848, 0.966]	2.95E-03	1	0	0	1
**97**	EFCAB14	0.900	[0.842, 0.962]	2.13E-03	0.941	[0.895, 0.989]	1.79E-02	0	0	0	0
**98**	SYNRG	0.898	[0.838, 0.963]	2.53E-03	0.936	[0.889, 0.985]	1.07E-02	1	0	0	1
**99**	SRSF5	0.895	[0.821, 0.976]	1.25E-02	0.934	[0.886, 0.984]	1.09E-02	1	0	0	1
**100**	LETM1	0.894	[0.82,0. 975]	1.15E-02	0.872	[0.814, 0.935]	1.51E-04	1	0	0	0
**101**	MACF1	0.892	[0.818, 0.973]	1.05E-02	0.905	[0.858, 0.956]	3.84E-04	1	0	0	0
**102**	NFATC3	0.892	[0.829, 0.959]	2.27E-03	0.914	[0.868, 0.962]	7.35E-04	1	0	0	0
**103**	BAZ1B	0.890	[0.817, 0.968]	6.97E-03	0.919	[0.866, 0.975]	5.67E-03	1	0	0	0

**104**	NOL9	0.887	[0.803, 0.98]	1.87E-02	0.902	[0.843, 0.964]	2.76E-03	1	0	0	0
**105**	TCF7	0.883	[0.801, 0.975]	1.36E-02	0.918	[0.857, 0.983]	1.49E-02	1	0	0	1
**106**	TIAM1	0.881	[0.79,0.983]	2.31E-02	0.911	[0.84,0.988]	2.44E-02	1	0	0	0
**107**	PAPD7	0.879	[0.799, 0.968]	8.74E-03	0.900	[0.841, 0.964]	2.68E-03	1	0	0	0
**108**	ZNF789	0.877	[0.791, 0.973]	1.35E-02	0.913	[0.844, 0.988]	2.43E-02	1	0	0	0
**109**	FNBP4	0.876	[0.781, 0.981]	2.25E-02	0.902	[0.84,0.968]	4.55E-03	1	0	0	0
**110**	SLC7A6OS	0.876	[0.783, 0.98]	2.06E-02	0.913	[0.851, 0.98]	1.21E-02	1	0	0	0
**111**	TBP	0.874	[0.808, 0.945]	8.48E-04	0.924	[0.877, 0.973]	3.20E-03	1	0	0	0
**112**	EP400	0.873	[0.787, 0.968]	1.04E-02	0.876	[0.815, 0.942]	4.10E-04	1	0	0	0
**113**	EML4	0.871	[0.774, 0.981]	2.35E-02	0.901	[0.834, 0.972]	7.67E-03	1	0	0	0
**114**	PDCD7	0.869	[0.797, 0.948]	1.75E-03	0.927	[0.871, 0.986]	1.64E-02	1	0	0	0
**115**	SLC7A6	0.866	[0.779, 0.962]	7.52E-03	0.904	[0.841, 0.973]	7.15E-03	1	0	0	0
**116**	EPM2AIP1	0.862	[0.785, 0.947]	2.18E-03	0.917	[0.851, 0.988]	2.38E-02	1	0	0	0
**117**	ABLIM1	0.862	[0.771, 0.963]	9.05E-03	0.907	[0.842, 0.977]	1.03E-02	1	0	0	1
**118**	RPL22	0.861	[0.781, 0.949]	2.84E-03	0.911	[0.846, 0.981]	1.39E-02	1	0	0	0
**119**	CD96	0.861	[0.766, 0.967]	1.19E-02	0.892	[0.817, 0.974]	1.12E-02	1	0	0	1
**120**	DDX27	0.860	[0.771, 0.96]	7.48E-03	0.926	[0.866, 0.99]	2.44E-02	1	0	0	0
**121**	CBFA2T2	0.860	[0.776, 0.953]	4.12E-03	0.906	[0.845, 0.97]	4.93E-03	1	0	0	1
**122**	ZSCAN22	0.855	[0.758, 0.964]	1.07E-02	0.871	[0.79,0.96]	5.61E-03	1	0	0	0
**123**	ST6GAL1	0.854	[0.774, 0.941]	1.66E-03	0.906	[0.839, 0.977]	1.09E-02	1	1	1	1
**124**	KMT2A	0.852	[0.762, 0.954]	5.63E-03	0.905	[0.844, 0.97]	4.90E-03	0	0	0	0
**125**	CBY1	0.852	[0.765, 0.949]	3.73E-03	0.925	[0.867, 0.985]	1.62E-02	1	0	0	0
**126**	OXNAD1	0.852	[0.746, 0.973]	1.83E-02	0.874	[0.787, 0.971]	1.20E-02	1	0	0	1
**127**	FAM102A	0.851	[0.743, 0.973]	1.90E-02	0.889	[0.809, 0.977]	1.43E-02	1	0	0	0
**128**	SRSF6	0.849	[0.767, 0.939]	1.69E-03	0.914	[0.848, 0.985]	1.89E-02	1	0	0	1
**129**	BZRAP1-AS1	0.849	[0.751, 0.959]	8.55E-03	0.887	[0.812, 0.969]	8.11E-03	0	0	0	0
**130**	PRKCQ	0.844	[0.747, 0.953]	6.68E-03	0.844	[0.767, 0.928]	5.49E-04	1	0	0	1
**131**	ATP8B2	0.841	[0.74,0.956]	8.38E-03	0.886	[0.813, 0.966]	6.25E-03	1	0	0	1
**132**	THOC1	0.836	[0.751, 0.931]	1.20E-03	0.858	[0.791, 0.931]	2.76E-04	1	0	0	1
**133**	PIK3R1	0.836	[0.748, 0.933]	1.62E-03	0.905	[0.842, 0.972]	6.45E-03	1	0	1	1
**134**	USP47	0.835	[0.726, 0.961]	1.22E-02	0.878	[0.806, 0.957]	3.26E-03	1	0	0	0
**135**	LOC93622	0.834	[0.755, 0.923]	4.91E-04	0.902	[0.834, 0.976]	1.04E-02	0	0	0	0
**136**	CEP95	0.832	[0.731, 0.946]	5.36E-03	0.872	[0.803, 0.946]	1.08E-03	1	0	0	0
**137**	BCL11B	0.828	[0.705, 0.971]	2.07E-02	0.865	[0.788, 0.949]	2.34E-03	1	0	0	0
**138**	TRIB2	0.827	[0.719, 0.952]	8.54E-03	0.871	[0.792, 0.957]	4.39E-03	1	0	0	0
**139**	LRRC39	0.825	[0.709, 0.96]	1.34E-02	0.855	[0.76,0.962]	9.44E-03	1	0	0	0
**140**	ARHGEF9	0.825	[0.707, 0.963]	1.49E-02	0.880	[0.798, 0.97]	1.05E-02	1	0	0	1

**141**	USP13	0.823	[0.724, 0.935]	3.02E-03	0.892	[0.808, 0.985]	2.36E-02	1	0	0	1
**142**	CD200R1	0.823	[0.699, 0.969]	1.94E-02	0.872	[0.776, 0.978]	2.00E-02	1	0	0	1
**143**	CD247	0.823	[0.728, 0.929]	1.87E-03	0.894	[0.814, 0.982]	1.91E-02	1	1	1	1
**144**	NOL8	0.823	[0.73,0.927]	1.54E-03	0.904	[0.833, 0.981]	1.61E-02	1	0	0	1
**145**	POU6F1	0.820	[0.698, 0.963]	1.57E-02	0.823	[0.725, 0.934]	2.78E-03	1	0	0	0
**146**	CFAP36	0.819	[0.717, 0.935]	3.44E-03	0.898	[0.818, 0.986]	2.44E-02	0	0	0	0
**147**	SMAD3	0.818	[0.733, 0.914]	4.37E-04	0.888	[0.822, 0.959]	2.60E-03	1	0	0	0
**148**	STAT4	0.818	[0.723, 0.924]	1.42E-03	0.872	[0.793, 0.96]	5.55E-03	1	0	1	1
**149**	LPAR5	0.816	[0.715, 0.93]	2.57E-03	0.879	[0.791, 0.977]	1.73E-02	1	0	0	0
**150**	AP3M2	0.815	[0.7,0.949]	8.63E-03	0.871	[0.791, 0.96]	5.43E-03	1	0	0	0
**151**	SAMD3	0.814	[0.706, 0.939]	4.89E-03	0.868	[0.772, 0.976]	1.78E-02	1	0	1	1
**152**	PRKCH	0.813	[0.73,0.904]	1.71E-04	0.879	[0.809, 0.955]	2.37E-03	1	1	1	1
**153**	MTSS1	0.807	[0.68,0.958]	1.46E-02	0.873	[0.775, 0.982]	2.43E-02	1	1	1	1
**154**	DYNC2H1	0.805	[0.689, 0.94]	6.51E-03	0.801	[0.687, 0.934]	4.89E-03	1	0	0	0
**155**	IQCH-AS1	0.803	[0.663, 0.972]	2.45E-02	0.837	[0.729, 0.96]	1.13E-02	0	0	0	0
**156**	NFATC2	0.801	[0.712, 0.902]	2.96E-04	0.878	[0.802, 0.961]	4.99E-03	1	1	1	1
**157**	TMEM194A	0.782	[0.664, 0.919]	3.16E-03	0.875	[0.781, 0.98]	2.15E-02	1	0	0	0
**158**	ERMP1	0.778	[0.655, 0.925]	4.65E-03	0.878	[0.789, 0.978]	1.86E-02	1	0	0	0
**159**	MORC4	0.762	[0.64,0.908]	2.54E-03	0.880	[0.79,0.98]	2.07E-02	1	0	0	1
**160**	KLRC4	0.744	[0.601, 0.921]	7.03E-03	0.711	[0.589, 0.859]	4.79E-04	1	0	0	0
**161**	GALNT12	0.741	[0.601, 0.914]	5.37E-03	0.834	[0.734, 0.948]	5.55E-03	1	0	0	1
**162**	LDLRAD4-AS1	0.738	[0.615, 0.885]	1.17E-03	0.822	[0.713, 0.947]	7.06E-03	0	0	0	0
**163**	RTTN	0.732	[0.631, 0.849]	5.45E-05	0.894	[0.815, 0.98]	1.76E-02	1	0	0	0
**164**	FBXO32	0.703	[0.547, 0.902]	5.91E-03	0.832	[0.724, 0.956]	9.83E-03	1	0	0	0
**165**	EPHA4	0.696	[0.534, 0.908]	7.83E-03	0.789	[0.661, 0.942]	9.04E-03	1	0	0	0

Table S6. List of 393 genes that are significantly differentially expressed across both studies using Fisher's chi squared test with FDR=10%. S = chi-squared statistics; P = *P*-value.

Rank	Symbol	S	P	FDR
**1**	S100A12	17.617	4.16E-07	5.23E-03
**2**	HP	17.302	5.60E-07	5.23E-03
**3**	TLR2	16.698	9.91E-07	6.16E-03
**4**	ATF7IP2	16.203	1.58E-06	6.54E-03
**5**	MGST1	16.093	1.75E-06	6.54E-03
**6**	CAMP	15.594	2.80E-06	8.66E-03
**7**	MARC1	15.226	3.96E-06	8.66E-03
**8**	CD63	15.204	4.04E-06	8.66E-03
**9**	PFKFB2	14.931	5.22E-06	8.66E-03
**10**	THOC1	14.921	5.27E-06	8.66E-03
**11**	CAPNS2	14.746	6.21E-06	8.66E-03
**12**	PRKCH	14.718	6.37E-06	8.66E-03
**13**	S100A6	14.695	6.51E-06	8.66E-03
**14**	RNASE1	14.674	6.64E-06	8.66E-03
**15**	CARD6	14.624	6.96E-06	8.66E-03
**16**	S100A9	14.508	7.76E-06	9.05E-03
**17**	PGLYRP1	14.378	8.76E-06	9.62E-03
**18**	RETN	14.173	1.06E-05	1.08E-02
**19**	SLPI	14.131	1.10E-05	1.08E-02
**20**	RTTN	13.858	1.42E-05	1.28E-02
**21**	CEBPA	13.849	1.44E-05	1.28E-02
**22**	P2RY8	13.785	1.52E-05	1.29E-02
**23**	SMAD3	13.688	1.67E-05	1.30E-02
**24**	MMP8	13.660	1.71E-05	1.30E-02
**25**	SFN	13.624	1.77E-05	1.30E-02
**26**	AAK1	13.597	1.82E-05	1.30E-02
**27**	RNASE2	13.529	1.93E-05	1.34E-02
**28**	NFATC2	13.426	2.13E-05	1.41E-02
**29**	KANSL3	13.397	2.19E-05	1.41E-02
**30**	NFATC3	13.302	2.39E-05	1.49E-02
**31**	LETM1	13.266	2.47E-05	1.49E-02
**32**	LOC100507507	13.045	3.04E-05	1.77E-02
**33**	TBP	12.816	3.75E-05	2.09E-02
**34**	PQLC1	12.803	3.80E-05	2.09E-02
**35**	IL13RA1	12.744	4.01E-05	2.14E-02
**36**	KLRC4	12.601	4.58E-05	2.38E-02
**37**	PRKCQ	12.516	4.96E-05	2.50E-02
**38**	PRORSD1P	12.463	5.21E-05	2.54E-02
**39**	MACF1	12.425	5.39E-05	2.54E-02
**40**	GRB10	12.400	5.52E-05	2.54E-02
**41**	LCN2	12.379	5.63E-05	2.54E-02
**42**	EP400	12.363	5.71E-05	2.54E-02
**43**	TNNT1	12.303	6.04E-05	2.56E-02
**44**	SORT1	12.276	6.19E-05	2.56E-02
**45**	ARL4C	12.275	6.20E-05	2.56E-02
**46**	ZNF345	12.232	6.45E-05	2.56E-02
**47**	LOC93622	12.190	6.70E-05	2.56E-02
**48**	TP53I3	12.187	6.72E-05	2.56E-02
**49**	PPP3CC	12.186	6.73E-05	2.56E-02

**50**	ASGR2	12.135	7.05E-05	2.63E-02
**51**	ECRP	12.081	7.41E-05	2.65E-02
**52**	MKNK1	12.065	7.52E-05	2.65E-02
**53**	CEP95	12.064	7.53E-05	2.65E-02
**54**	RNASE3	11.996	8.02E-05	2.77E-02
**55**	MGC27345	11.818	9.44E-05	3.20E-02
**56**	KLHL4	11.755	1.00E-04	3.28E-02
**57**	STAT4	11.753	1.00E-04	3.28E-02
**58**	ZDHHC19	11.724	1.03E-04	3.31E-02
**59**	LDLRAD4-AS1	11.708	1.05E-04	3.31E-02
**60**	SMAD7	11.690	1.06E-04	3.31E-02
**61**	XPNPEP1	11.660	1.09E-04	3.31E-02
**62**	NLRP3	11.641	1.11E-04	3.31E-02
**63**	GRIA1	11.637	1.12E-04	3.31E-02
**64**	CAPG	11.617	1.14E-04	3.32E-02
**65**	TDRD9	11.545	1.22E-04	3.45E-02
**66**	ATOX1	11.539	1.22E-04	3.45E-02
**67**	LYRM2	11.525	1.24E-04	3.45E-02
**68**	SLC47A1	11.496	1.27E-04	3.48E-02
**69**	NRG1	11.474	1.30E-04	3.48E-02
**70**	PIK3R1	11.468	1.30E-04	3.48E-02
**71**	TSHZ3	11.375	1.42E-04	3.72E-02
**72**	BCL7A	11.356	1.45E-04	3.72E-02
**73**	AGTRAP	11.316	1.50E-04	3.72E-02
**74**	PLBD1	11.315	1.50E-04	3.72E-02
**75**	CHIC1	11.307	1.51E-04	3.72E-02
**76**	TSPO	11.305	1.52E-04	3.72E-02
**77**	MAFG	11.251	1.59E-04	3.74E-02
**78**	TSEN34	11.245	1.60E-04	3.74E-02
**79**	GABARAP	11.227	1.63E-04	3.74E-02
**80**	CD163	11.220	1.64E-04	3.74E-02
**81**	RASA3	11.218	1.64E-04	3.74E-02
**82**	NR2F6	11.216	1.64E-04	3.74E-02
**83**	DDB2	11.183	1.69E-04	3.81E-02
**84**	PSMB3	11.158	1.73E-04	3.85E-02
**85**	CKLF	11.115	1.80E-04	3.85E-02
**86**	OSCAR	11.115	1.80E-04	3.85E-02
**87**	DCC	11.097	1.83E-04	3.85E-02
**88**	MROH6	11.096	1.83E-04	3.85E-02
**89**	GPR84	11.095	1.84E-04	3.85E-02
**90**	C10orf11	11.053	1.91E-04	3.96E-02
**91**	TNIK	11.029	1.95E-04	4.00E-02
**92**	SV2B	10.989	2.02E-04	4.11E-02
**93**	LGALS1	10.964	2.07E-04	4.16E-02
**94**	MYO19	10.950	2.10E-04	4.16E-02
**95**	CAPRIN2	10.936	2.13E-04	4.17E-02
**96**	RBBP4	10.927	2.14E-04	4.17E-02
**97**	ST6GAL1	10.915	2.17E-04	4.17E-02
**98**	TMEM11	10.871	2.26E-04	4.30E-02
**99**	CBFA2T2	10.803	2.40E-04	4.53E-02
**100**	INPP5A	10.792	2.43E-04	4.53E-02
**101**	BTRC	10.775	2.46E-04	4.55E-02
**102**	LINC01503	10.708	2.62E-04	4.72E-02
**103**	MRPS36	10.698	2.64E-04	4.72E-02

**104**	ATP6AP1	10.689	2.66E-04	4.72E-02
**105**	ACOT13	10.676	2.70E-04	4.72E-02
**106**	AFAP1L2	10.667	2.72E-04	4.72E-02
**107**	CTSH	10.667	2.72E-04	4.72E-02
**108**	PAPD7	10.661	2.73E-04	4.72E-02
**109**	NOL8	10.603	2.88E-04	4.93E-02
**110**	UBTD1	10.593	2.91E-04	4.93E-02
**111**	SYNRG	10.514	3.13E-04	5.13E-02
**112**	TPST1	10.512	3.13E-04	5.13E-02
**113**	METTL20	10.509	3.14E-04	5.13E-02
**114**	MCEMP1	10.509	3.14E-04	5.13E-02
**115**	KMT2A	10.499	3.17E-04	5.13E-02
**116**	PFKFB4	10.492	3.19E-04	5.13E-02
**117**	HK3	10.468	3.26E-04	5.19E-02
**118**	PDCD7	10.461	3.28E-04	5.19E-02
**119**	TRG-AS1	10.444	3.33E-04	5.23E-02
**120**	GALNT12	10.421	3.40E-04	5.27E-02
**121**	IL17RA	10.389	3.50E-04	5.27E-02
**122**	CHIT1	10.389	3.50E-04	5.27E-02
**123**	FAM134C	10.386	3.52E-04	5.27E-02
**124**	LRIG1	10.372	3.56E-04	5.27E-02
**125**	LMX1B	10.371	3.56E-04	5.27E-02
**126**	PTS	10.362	3.59E-04	5.27E-02
**127**	DYNC2H1	10.355	3.62E-04	5.27E-02
**128**	SRSF6	10.352	3.63E-04	5.27E-02
**129**	PYCARD	10.333	3.69E-04	5.27E-02
**130**	PYHIN1	10.332	3.69E-04	5.27E-02
**131**	ANO10	10.329	3.70E-04	5.27E-02
**132**	ADAMDEC1	10.314	3.75E-04	5.29E-02
**133**	GPR171	10.310	3.77E-04	5.29E-02
**134**	RBM15B	10.267	3.92E-04	5.40E-02
**135**	QRICH1	10.266	3.92E-04	5.40E-02
**136**	FOXP1	10.259	3.94E-04	5.40E-02
**137**	ABCD4	10.254	3.96E-04	5.40E-02
**138**	CD247	10.241	4.01E-04	5.42E-02
**139**	TRIB2	10.191	4.20E-04	5.58E-02
**140**	EFCAB14	10.178	4.25E-04	5.58E-02
**141**	GSTO1	10.178	4.25E-04	5.58E-02
**142**	GNG5	10.151	4.35E-04	5.58E-02
**143**	CTSD	10.142	4.39E-04	5.58E-02
**144**	RPL22	10.142	4.39E-04	5.58E-02
**145**	BAZ1B	10.139	4.40E-04	5.58E-02
**146**	SF3A1	10.137	4.41E-04	5.58E-02
**147**	USP47	10.129	4.44E-04	5.58E-02
**148**	NDUFB6	10.121	4.47E-04	5.58E-02
**149**	TCN1	10.116	4.49E-04	5.58E-02
**150**	CLEC4A	10.107	4.53E-04	5.58E-02
**151**	RTFDC1	10.107	4.53E-04	5.58E-02
**152**	ERGIC1	10.104	4.54E-04	5.58E-02
**153**	KRT12	10.078	4.65E-04	5.67E-02
**154**	C14orf28	10.044	4.80E-04	5.79E-02
**155**	POU6F1	10.040	4.81E-04	5.79E-02
**156**	PTRHD1	10.031	4.85E-04	5.79E-02
**157**	LPAR5	10.018	4.91E-04	5.79E-02

**158**	MLKL	10.007	4.96E-04	5.79E-02
**159**	LMO2	10.006	4.97E-04	5.79E-02
**160**	DOK1	10.004	4.97E-04	5.79E-02
**161**	BPI	10.000	4.99E-04	5.79E-02
**162**	DEFA4	9.975	5.11E-04	5.83E-02
**163**	METAP1	9.973	5.12E-04	5.83E-02
**164**	AP3M2	9.969	5.14E-04	5.83E-02
**165**	ATP6V0E1	9.966	5.15E-04	5.83E-02
**166**	CRISP3	9.948	5.24E-04	5.83E-02
**167**	KLRB1	9.947	5.24E-04	5.83E-02
**168**	SEMA4A	9.946	5.25E-04	5.83E-02
**169**	TRPM2	9.935	5.30E-04	5.83E-02
**170**	BCL11B	9.932	5.31E-04	5.83E-02
**171**	MFSD6	9.915	5.39E-04	5.88E-02
**172**	MBTPS1	9.908	5.43E-04	5.88E-02
**173**	RORA	9.877	5.58E-04	5.88E-02
**174**	REPS1	9.875	5.60E-04	5.88E-02
**175**	NOL9	9.871	5.62E-04	5.88E-02
**176**	GON4L	9.866	5.64E-04	5.88E-02
**177**	EPM2AIP1	9.865	5.65E-04	5.88E-02
**178**	TTC23	9.858	5.68E-04	5.88E-02
**179**	ATP8B2	9.857	5.68E-04	5.88E-02
**180**	CCDC65	9.856	5.69E-04	5.88E-02
**181**	MORC4	9.854	5.70E-04	5.88E-02
**182**	ADM	9.848	5.74E-04	5.88E-02
**183**	SLC7A6	9.831	5.82E-04	5.94E-02
**184**	IL7R	9.823	5.86E-04	5.95E-02
**185**	FGD4	9.814	5.91E-04	5.97E-02
**186**	MORF4L2-AS1	9.778	6.11E-04	6.11E-02
**187**	GPATCH8	9.775	6.13E-04	6.11E-02
**188**	FBXO32	9.753	6.25E-04	6.20E-02
**189**	BRF1	9.746	6.29E-04	6.21E-02
**190**	LOXL3	9.731	6.37E-04	6.26E-02
**191**	ZSCAN22	9.717	6.46E-04	6.29E-02
**192**	CBY1	9.714	6.47E-04	6.29E-02
**193**	CYSTM1	9.698	6.57E-04	6.33E-02
**194**	GZMK	9.696	6.58E-04	6.33E-02
**195**	LHFPL2	9.682	6.66E-04	6.35E-02
**196**	BRIX1	9.681	6.67E-04	6.35E-02
**197**	CD48	9.658	6.81E-04	6.45E-02
**198**	CLDND1	9.649	6.87E-04	6.47E-02
**199**	IFNGR2	9.638	6.94E-04	6.47E-02
**200**	METTL14	9.629	7.00E-04	6.47E-02
**201**	ZDHHC12	9.623	7.03E-04	6.47E-02
**202**	CEACAM6	9.620	7.05E-04	6.47E-02
**203**	TFEB	9.614	7.09E-04	6.47E-02
**204**	DLGAP3	9.605	7.14E-04	6.47E-02
**205**	DUSP3	9.601	7.17E-04	6.47E-02
**206**	IMPDH1	9.597	7.20E-04	6.47E-02
**207**	RAD1	9.597	7.20E-04	6.47E-02
**208**	TMEM194A	9.595	7.21E-04	6.47E-02
**209**	BZRAP1-AS1	9.577	7.33E-04	6.55E-02
**210**	EPHA4	9.556	7.47E-04	6.64E-02
**211**	USP13	9.548	7.53E-04	6.65E-02

**212**	PNPLA2	9.545	7.55E-04	6.65E-02
**213**	SMARCD3	9.525	7.68E-04	6.73E-02
**214**	EZR	9.456	8.18E-04	7.13E-02
**215**	TSPAN1	9.445	8.26E-04	7.16E-02
**216**	S1PR1	9.442	8.29E-04	7.16E-02
**217**	RNF135	9.427	8.39E-04	7.21E-02
**218**	MAP2K6	9.410	8.53E-04	7.21E-02
**219**	SH2B2	9.405	8.56E-04	7.21E-02
**220**	SERPING1	9.403	8.58E-04	7.21E-02
**221**	PELO	9.391	8.67E-04	7.21E-02
**222**	PCOLCE2	9.391	8.68E-04	7.21E-02
**223**	TTC39C	9.388	8.70E-04	7.21E-02
**224**	CFAP36	9.386	8.71E-04	7.21E-02
**225**	ATG7	9.384	8.73E-04	7.21E-02
**226**	PLAUR	9.383	8.74E-04	7.21E-02
**227**	CLEC4E	9.370	8.84E-04	7.27E-02
**228**	ERMP1	9.358	8.93E-04	7.31E-02
**229**	SAMD3	9.348	9.02E-04	7.32E-02
**230**	RAB32	9.346	9.03E-04	7.32E-02
**231**	TXN	9.343	9.06E-04	7.32E-02
**232**	ZNF566	9.333	9.14E-04	7.33E-02
**233**	LOC101928140	9.329	9.17E-04	7.33E-02
**234**	USPL1	9.320	9.25E-04	7.33E-02
**235**	EXOC2	9.316	9.28E-04	7.33E-02
**236**	PLTP	9.309	9.34E-04	7.33E-02
**237**	ZNF438	9.306	9.36E-04	7.33E-02
**238**	C12orf79	9.298	9.43E-04	7.33E-02
**239**	ARHGEF7	9.298	9.43E-04	7.33E-02
**240**	TGFBR3	9.290	9.50E-04	7.33E-02
**241**	LAMTOR5	9.290	9.50E-04	7.33E-02
**242**	MAPK14	9.290	9.50E-04	7.33E-02
**243**	ABLIM1	9.282	9.57E-04	7.35E-02
**244**	PLIN3	9.265	9.72E-04	7.43E-02
**245**	ZBTB4	9.251	9.85E-04	7.50E-02
**246**	ACSL5	9.239	9.95E-04	7.53E-02
**247**	LY9	9.236	9.97E-04	7.53E-02
**248**	TULP4	9.233	1.00E-03	7.53E-02
**249**	GDE1	9.226	1.01E-03	7.55E-02
**250**	MYCBPAP	9.219	1.01E-03	7.56E-02
**251**	ROPN1L	9.213	1.02E-03	7.56E-02
**252**	CSRP3	9.211	1.02E-03	7.56E-02
**253**	KIAA1656	9.194	1.04E-03	7.61E-02
**254**	GADD45A	9.192	1.04E-03	7.61E-02
**255**	ALAS1	9.188	1.04E-03	7.61E-02
**256**	FNBP4	9.186	1.04E-03	7.61E-02
**257**	CHCHD1	9.161	1.07E-03	7.75E-02
**258**	TARSL2	9.157	1.07E-03	7.75E-02
**259**	DMRTB1	9.152	1.08E-03	7.75E-02
**260**	LDOC1L	9.140	1.09E-03	7.77E-02
**261**	CEACAM8	9.130	1.10E-03	7.77E-02
**262**	CLEC1B	9.128	1.10E-03	7.77E-02
**263**	ARID5B	9.124	1.10E-03	7.77E-02
**264**	CAMKK2	9.121	1.11E-03	7.77E-02
**265**	HERC2	9.120	1.11E-03	7.77E-02

**266**	CEACAM4	9.120	1.11E-03	7.77E-02
**267**	TBCC	9.112	1.12E-03	7.80E-02
**268**	TLR5	9.105	1.12E-03	7.82E-02
**269**	ZHX3	9.081	1.15E-03	7.90E-02
**270**	GALE	9.081	1.15E-03	7.90E-02
**271**	TMEM9	9.081	1.15E-03	7.90E-02
**272**	OSBP	9.075	1.15E-03	7.91E-02
**273**	CPA2	9.061	1.17E-03	7.99E-02
**274**	MGST2	9.052	1.18E-03	7.99E-02
**275**	HADHB	9.047	1.18E-03	7.99E-02
**276**	NLRC4	9.045	1.19E-03	7.99E-02
**277**	CLSTN1	9.042	1.19E-03	7.99E-02
**278**	SPINK2	9.041	1.19E-03	7.99E-02
**279**	C1orf21	9.034	1.20E-03	8.01E-02
**280**	HIST1H2BK	9.030	1.20E-03	8.01E-02
**281**	NEU1	9.023	1.21E-03	8.03E-02
**282**	BRI3	9.001	1.23E-03	8.14E-02
**283**	DHRS3	9.000	1.23E-03	8.14E-02
**284**	EOMES	8.994	1.24E-03	8.14E-02
**285**	A1BG	8.989	1.25E-03	8.14E-02
**286**	KIAA1143	8.988	1.25E-03	8.14E-02
**287**	LRRC39	8.977	1.26E-03	8.19E-02
**288**	PHF7	8.972	1.27E-03	8.20E-02
**289**	SYN2	8.962	1.28E-03	8.24E-02
**290**	A2M	8.955	1.29E-03	8.27E-02
**291**	LINC00330	8.944	1.30E-03	8.30E-02
**292**	SETBP1	8.944	1.30E-03	8.30E-02
**293**	CD96	8.924	1.32E-03	8.40E-02
**294**	MXD3	8.923	1.32E-03	8.40E-02
**295**	CLTCL1	8.914	1.33E-03	8.44E-02
**296**	BTBD9	8.906	1.34E-03	8.44E-02
**297**	MDN1	8.906	1.34E-03	8.44E-02
**298**	SRSF5	8.894	1.36E-03	8.46E-02
**299**	TNFRSF25	8.893	1.36E-03	8.46E-02
**300**	WIPI1	8.892	1.36E-03	8.46E-02
**301**	TTC36	8.881	1.37E-03	8.47E-02
**302**	MMP9	8.873	1.38E-03	8.47E-02
**303**	ZBTB40	8.870	1.39E-03	8.47E-02
**304**	ASXL1	8.868	1.39E-03	8.47E-02
**305**	GNA15	8.867	1.39E-03	8.47E-02
**306**	CLIC1	8.865	1.39E-03	8.47E-02
**307**	PRPF38A	8.861	1.40E-03	8.47E-02
**308**	SDF2	8.858	1.40E-03	8.47E-02
**309**	GPR18	8.858	1.40E-03	8.47E-02
**310**	ROMO1	8.843	1.42E-03	8.56E-02
**311**	EIF1AD	8.833	1.43E-03	8.61E-02
**312**	OIP5-AS1	8.824	1.45E-03	8.65E-02
**313**	LRG1	8.819	1.45E-03	8.66E-02
**314**	ARHGEF5	8.806	1.47E-03	8.70E-02
**315**	MDC1	8.804	1.47E-03	8.70E-02
**316**	LTB4R	8.803	1.47E-03	8.70E-02
**317**	ABCC2	8.796	1.48E-03	8.73E-02
**318**	OTUD3	8.778	1.51E-03	8.82E-02
**319**	FANCD2OS	8.777	1.51E-03	8.82E-02

**320**	NOLC1	8.768	1.52E-03	8.86E-02
**321**	PPM1K	8.763	1.53E-03	8.86E-02
**322**	ARHGEF9	8.761	1.53E-03	8.86E-02
**323**	KCNH1	8.745	1.55E-03	8.97E-02
**324**	S1PR3	8.732	1.57E-03	9.04E-02
**325**	RSPH9	8.722	1.58E-03	9.10E-02
**326**	NUP205	8.714	1.60E-03	9.14E-02
**327**	BST1	8.708	1.60E-03	9.16E-02
**328**	FBLN1	8.690	1.63E-03	9.24E-02
**329**	PDE4D	8.689	1.63E-03	9.24E-02
**330**	SQRDL	8.688	1.63E-03	9.24E-02
**331**	PSTPIP2	8.676	1.65E-03	9.31E-02
**332**	TWF2	8.673	1.66E-03	9.31E-02
**333**	NAIP	8.664	1.67E-03	9.35E-02
**334**	GTF3A	8.652	1.69E-03	9.41E-02
**335**	DAD1	8.651	1.69E-03	9.41E-02
**336**	DHX33	8.642	1.70E-03	9.45E-02
**337**	KIAA0020	8.638	1.71E-03	9.46E-02
**338**	GATA3	8.629	1.72E-03	9.51E-02
**339**	EML4	8.622	1.73E-03	9.52E-02
**340**	DPP4	8.621	1.73E-03	9.52E-02
**341**	DDX27	8.610	1.75E-03	9.56E-02
**342**	PHC2	8.605	1.76E-03	9.56E-02
**343**	PGM1	8.603	1.76E-03	9.56E-02
**344**	ATP1A1	8.600	1.77E-03	9.56E-02
**345**	CD5	8.600	1.77E-03	9.56E-02
**346**	FNDC8	8.595	1.78E-03	9.58E-02
**347**	GLA	8.591	1.78E-03	9.58E-02
**348**	GSN	8.579	1.80E-03	9.61E-02
**349**	TCEB2	8.577	1.80E-03	9.61E-02
**350**	SLC2A4RG	8.575	1.81E-03	9.61E-02
**351**	BLOC1S1	8.575	1.81E-03	9.61E-02
**352**	PNMA1	8.569	1.82E-03	9.63E-02
**353**	USP32	8.566	1.82E-03	9.63E-02
**354**	PIEZO1	8.563	1.83E-03	9.63E-02
**355**	SUPT16H	8.555	1.84E-03	9.65E-02
**356**	POR	8.549	1.85E-03	9.65E-02
**357**	ACVR1B	8.549	1.85E-03	9.65E-02
**358**	SYTL2	8.548	1.85E-03	9.65E-02
**359**	GAPDH	8.543	1.86E-03	9.67E-02
**360**	RTN4RL2	8.537	1.87E-03	9.68E-02
**361**	CDK15	8.536	1.87E-03	9.68E-02
**362**	CCDC84	8.527	1.89E-03	9.70E-02
**363**	CNOT6L	8.526	1.89E-03	9.70E-02
**364**	NAA38	8.521	1.90E-03	9.70E-02
**365**	ZNF331	8.520	1.90E-03	9.70E-02
**366**	GM2A	8.518	1.90E-03	9.70E-02
**367**	LOC101927040	8.507	1.92E-03	9.77E-02
**368**	RBP7	8.502	1.93E-03	9.77E-02
**369**	LINC00269	8.499	1.93E-03	9.77E-02
**370**	TCF7	8.498	1.94E-03	9.77E-02
**371**	RPS19BP1	8.490	1.95E-03	9.80E-02
**372**	GCHFR	8.488	1.95E-03	9.80E-02
**373**	REPS2	8.476	1.98E-03	9.88E-02

**374**	AHNAK	8.469	1.99E-03	9.89E-02
**375**	SHKBP1	8.469	1.99E-03	9.89E-02
**376**	CABLES2	8.464	2.00E-03	9.89E-02
**377**	TBXAS1	8.453	2.02E-03	9.89E-02
**378**	UPB1	8.453	2.02E-03	9.89E-02
**379**	CECR6	8.450	2.02E-03	9.89E-02
**380**	ELP2	8.449	2.02E-03	9.89E-02
**381**	C1RL	8.448	2.03E-03	9.89E-02
**382**	AIRE	8.439	2.04E-03	9.89E-02
**383**	C1orf127	8.433	2.05E-03	9.89E-02
**384**	TSHZ1	8.432	2.06E-03	9.89E-02
**385**	OXNAD1	8.424	2.07E-03	9.89E-02
**386**	SULT1B1	8.423	2.07E-03	9.89E-02
**387**	ALOX5	8.423	2.07E-03	9.89E-02
**388**	GUCY2D	8.423	2.07E-03	9.89E-02
**389**	SARM1	8.421	2.07E-03	9.89E-02
**390**	CATIP-AS1	8.418	2.08E-03	9.89E-02
**391**	DNAJC27	8.418	2.08E-03	9.89E-02
**392**	KCNE1	8.418	2.08E-03	9.89E-02
**393**	SLC26A8	8.417	2.08E-03	9.89E-02

Table S7. Enrichment analysis of genes differentially expressed in both GSK-HiTDiP and Janssen-BRC studies (Table S3). The top 10 gene ontology (GO) terms for Biological Processes (BP) are ranked according to their *P*-values from Fisher's exact test for significant enrichment. Bonferroni correction specifies a P-value threshold of P = $4.94x10^{-6}$ to achieve significance at q<0.05. Such a correction is well-known to be too stringent given that many of the GO terms are correlated, however the top 9 terms reported here remain significant even under Bonferroni correction.

Rank	GO.ID	Term	Annotated	Significant	Expected	Fisher
**1**	GO:0002376	immune system process	2149	52	21.63	3.0e-10
**2**	GO:0006955	immune response	1310	37	13.19	4.8e-09
**3**	GO:0042742	defense response to bacterium	164	13	1.65	1.1e-08
**4**	GO:0009617	response to bacterium	405	19	4.08	2.4e-08
**5**	GO:0043207	response to external biotic stimulus	651	22	6.55	5.6e-07
**6**	GO:0051707	response to other organism	651	22	6.55	5.6e-07
**7**	GO:0009607	response to biotic stimulus	680	22	6.84	1.2e-06
**8**	GO:0050832	defense response to fungus	21	5	0.21	1.7e-06
**9**	GO:0098542	defense response to other organism	351	15	3.53	2.5e-06
**10**	GO:0006952	defense response	1372	31	13.81	1.3e-05

Table S8. List of the 150 genes among the 165 genes in Table S5 that overlap with the microarray platform used in the NESDA study. The table displays fold-changes (FC) for the GSK-HiTDIP, Janssen-BRC and NESDA studies, as well as *P*-values (P) and false discovery rate estimates (FDR) for the NESDA study. The concordance of fold-changes is also provided (1 encodes concordance whereas 0 encodes non-concordance).

Rank	Symbol	HiTDIP FC	BRC FC	NESDA FC	Concordant	NESDA P	NESDA FDR
**1**	PRKCH	0.813	0.879	0.789	1	4.85e-05	2.95E-02
**2**	MTSS1	0.807	0.873	0.774	1	1.13E-04	3.64E-02
**3**	NFATC2	0.801	0.878	0.799	1	2.88E-04	4.05E-02
**4**	PGLYRP1	1.466	1.439	1.254	1	2.95E-04	4.05E-02
**5**	MAPK14	1.189	1.103	1.250	1	3e-04	4.06E-02
**6**	ST6GAL1	0.854	0.906	0.811	1	3.37E-04	4.20E-02
**7**	CD247	0.823	0.894	0.809	1	3.72E-04	4.34E-02
**8**	SLPI	1.586	1.546	1.237	1	8.91E-04	5.51E-02
**9**	SFN	1.202	1.183	1.219	1	1.01E-03	5.70E-02
**10**	ZBTB4	0.928	0.931	0.824	1	1.06E-03	5.70E-02
**11**	S100A8	1.127	1.080	1.207	1	1.08E-03	5.70E-02
**12**	ATG7	1.292	1.140	1.236	1	1.17E-03	5.77E-02
**13**	NRG1	1.570	1.384	1.237	1	1.18E-03	5.77E-02
**14**	PIK3R1	0.836	0.905	0.840	1	2.19E-03	7.22E-02
**15**	MMP9	1.500	1.343	1.220	1	2.28E-03	7.38E-02
**16**	SAMD3	0.814	0.868	0.830	1	3.10E-03	8.10E-02
**17**	STAT4	0.818	0.872	0.843	1	4.07E-03	9.13E-02
**18**	MCU	1.125	1.076	1.197	1	4.64E-03	9.49E-02
**19**	SYNRG	0.898	0.936	0.849	1	5.76E-03	1.01E-01
**20**	CARD6	1.209	1.225	1.192	1	5.86E-03	1.01E-01
**21**	SRSF5	0.895	0.934	0.868	1	5.91E-03	1.01E-01
**22**	CD200R1	0.823	0.872	0.835	1	6.42E-03	1.03E-01
**23**	GDE1	1.160	1.119	1.181	1	9.23E-03	1.19E-01
**24**	GALNT12	0.741	0.834	0.850	1	1.08E-02	1.24E-01
**25**	CD96	0.861	0.892	0.856	1	1.23E-02	1.31E-01
**26**	S100A9	1.208	1.133	1.170	1	1.24E-02	1.32E-01
**27**	TCF7	0.883	0.918	0.868	1	1.24E-02	1.32E-01
**28**	SRSF6	0.849	0.914	0.856	1	1.29E-02	1.34E-01
**29**	ATP8B2	0.841	0.886	0.865	1	1.80E-02	1.49E-01
**30**	CAMP	1.432	1.571	1.162	1	1.87E-02	1.51E-01
**31**	CBFA2T2	0.860	0.906	1.163	0	1.88E-02	1.51E-01
**32**	SULT1B1	1.302	1.239	1.155	1	1.98E-02	1.55E-01
**33**	HK3	1.281	1.162	1.145	1	2.18E-02	1.59E-01
**34**	THOC1	0.836	0.858	0.877	1	2.25E-02	1.61E-01
**35**	REPS1	0.902	0.905	0.868	1	2.25E-02	1.61E-01
**36**	ARHGEF9	0.825	0.880	0.865	1	2.25E-02	1.61E-01
**37**	OXNAD1	0.852	0.874	0.873	1	2.49E-02	1.67E-01
**38**	NAA38	1.141	1.103	0.874	0	2.73E-02	1.74E-01
**39**	S100A12	1.532	1.295	1.142	1	3.00E-02	1.80E-01
**40**	NOL8	0.823	0.904	0.875	1	3.03E-02	1.80E-01
**41**	USP13	0.823	0.892	0.873	1	3.17E-02	1.83E-01
**42**	PRKCQ	0.844	0.844	0.871	1	3.20E-02	1.84E-01
**43**	ABLIM1	0.862	0.907	0.892	1	3.47E-02	1.90E-01
**44**	MORC4	0.762	0.880	1.148	0	3.85E-02	1.96E-01
**45**	PLEKHN1	1.145	1.103	1.135	1	4.02E-02	2.00E-01
**46**	CKLF	1.156	1.158	1.121	1	4.38E-02	2.06E-01

**47**	MGST1	1.323	1.226	1.142	1	4.43E-02	2.07E-01
**48**	FAM102A	0.851	0.889	0.882	1	4.52E-02	2.08E-01
**49**	LETM1	0.894	0.872	1.135	0	4.66E-02	2.10E-01
**50**	OSBP	0.915	0.943	0.880	1	4.73E-02	2.12E-01
**51**	PARD6A	1.111	1.097	1.132	1	4.75E-02	2.12E-01
**52**	SMAD3	0.818	0.888	0.890	1	5.23E-02	2.20E-01
**53**	RPL22	0.861	0.911	0.895	1	5.39E-02	2.23E-01
**54**	KLHL4	1.161	1.113	1.134	1	5.70E-02	2.29E-01
**55**	HP	1.779	1.712	1.129	1	5.84E-02	2.31E-01
**56**	CRISP3	1.662	1.714	1.133	1	5.88E-02	2.31E-01
**57**	LPAR5	0.816	0.879	0.887	1	6.02E-02	2.33E-01
**58**	TRIB2	0.827	0.871	0.889	1	6.41E-02	2.38E-01
**59**	USP47	0.835	0.878	0.901	1	6.65E-02	2.42E-01
**60**	MKNK1	1.248	1.100	1.124	1	6.80E-02	2.45E-01
**61**	TBCC	1.126	1.094	0.887	0	6.91E-02	2.47E-01
**62**	EML4	0.871	0.901	0.899	1	6.99E-02	2.48E-01
**63**	GPR84	1.435	1.328	1.125	1	7.47E-02	2.53E-01
**64**	PYCARD	1.240	1.135	1.101	1	7.54E-02	2.54E-01
**65**	NFATC3	0.892	0.914	0.899	1	8.26E-02	2.66E-01
**66**	TSPO	1.224	1.153	1.099	1	8.56E-02	2.69E-01
**67**	PELO	1.178	1.168	1.114	1	8.66E-02	2.71E-01
**68**	CAPG	1.268	1.179	1.110	1	9.08E-02	2.75E-01
**69**	ASGR2	1.322	1.285	1.111	1	9.40E-02	2.80E-01
**70**	KLRC4	0.744	0.711	0.901	1	1.03E-01	2.89E-01
**71**	BPI	1.796	1.448	1.108	1	1.03E-01	2.89E-01
**72**	IMPDH1	1.250	1.121	1.095	1	1.06E-01	2.93E-01
**73**	MXD3	1.163	1.109	1.108	1	1.09E-01	2.96E-01
**74**	LGALS1	1.226	1.167	0.909	0	1.12E-01	2.99E-01
**75**	PREX2	1.128	1.089	1.104	1	1.16E-01	3.05E-01
**76**	ADM	1.177	1.254	1.104	1	1.18E-01	3.07E-01
**77**	ROPN1L	1.334	1.262	1.106	1	1.20E-01	3.09E-01
**78**	QRICH1	0.913	0.925	0.919	1	1.26E-01	3.16E-01
**79**	PDCD7	0.869	0.927	1.096	0	1.28E-01	3.19E-01
**80**	ERMP1	0.778	0.878	1.101	0	1.30E-01	3.20E-01
**81**	SV2B	1.198	1.126	0.904	0	1.32E-01	3.23E-01
**82**	EPHA4	0.696	0.789	0.906	1	1.33E-01	3.24E-01
**83**	TWF2	1.138	1.101	1.088	1	1.34E-01	3.25E-01
**84**	EP400	0.873	0.876	0.914	1	1.35E-01	3.26E-01
**85**	TLR5	1.294	1.197	1.101	1	1.37E-01	3.28E-01
**86**	SLC7A6	0.866	0.904	0.918	1	1.38E-01	3.29E-01
**87**	SLC7A6OS	0.876	0.913	0.917	1	1.40E-01	3.31E-01
**88**	GALE	1.121	1.105	1.095	1	1.42E-01	3.33E-01
**89**	FNBP4	0.876	0.902	0.921	1	1.45E-01	3.36E-01
**90**	TMEM194A	0.782	0.875	0.909	1	1.47E-01	3.39E-01
**91**	RNASE1	1.452	1.523	0.913	0	1.66E-01	3.56E-01
**92**	ASXL1	0.906	0.922	0.920	1	1.83E-01	3.71E-01
**93**	MACF1	0.892	0.905	0.932	1	1.96E-01	3.81E-01
**94**	TBP	0.874	0.924	0.921	1	1.96E-01	3.81E-01
**95**	TSEN34	1.262	1.147	1.081	1	2.03E-01	3.87E-01
**96**	PAPD7	0.879	0.900	0.929	1	2.06E-01	3.90E-01
**97**	ZSCAN22	0.855	0.871	0.920	1	2.15E-01	3.98E-01
**98**	LCN2	2.118	1.605	1.078	1	2.31E-01	4.13E-01
**99**	BRI3	1.170	1.118	1.083	1	2.33E-01	4.15E-01
**100**	LRRC39	0.825	0.855	0.925	1	2.40E-01	4.22E-01

**101**	FBXO32	0.703	0.832	0.924	1	2.40E-01	4.22E-01
**102**	CEACAM6	1.631	1.602	1.080	1	2.49E-01	4.30E-01
**103**	POU6F1	0.820	0.823	0.930	1	2.56E-01	4.37E-01
**104**	CEACAM8	1.916	1.687	1.075	1	2.57E-01	4.37E-01
**105**	RNASE2	1.564	1.368	0.931	0	2.70E-01	4.48E-01
**106**	CPNE2	1.226	1.137	1.068	1	2.71E-01	4.49E-01
**107**	PQLC1	1.200	1.148	0.937	0	2.93E-01	4.67E-01
**108**	TMEM120A	1.184	1.147	1.058	1	2.93E-01	4.67E-01
**109**	CYSTM1	1.228	1.250	1.067	1	2.95E-01	4.68E-01
**110**	GABARAP	1.081	1.045	1.057	1	2.98E-01	4.69E-01
**111**	MMP8	1.972	1.902	1.066	1	3.01E-01	4.72E-01
**112**	CLEC4A	1.191	1.139	1.064	1	3.04E-01	4.74E-01
**113**	RAB32	1.218	1.128	1.066	1	3.08E-01	4.78E-01
**114**	TP53I3	1.267	1.234	1.069	1	3.15E-01	4.84E-01
**115**	BAZ1B	0.890	0.919	0.940	1	3.23E-01	4.91E-01
**116**	HIST1H2BK	1.238	1.132	0.945	0	3.35E-01	5.00E-01
**117**	GNG5	1.110	1.049	0.949	0	3.38E-01	5.02E-01
**118**	DCC	1.085	1.137	1.065	1	3.38E-01	5.02E-01
**119**	TXN	1.196	1.149	1.062	1	3.47E-01	5.10E-01
**120**	TFEB	1.203	1.146	1.058	1	3.53E-01	5.14E-01
**121**	MAP2K6	1.208	1.150	1.061	1	3.74E-01	5.32E-01
**122**	CTSH	1.215	1.157	0.946	0	3.83E-01	5.39E-01
**123**	CBY1	0.852	0.925	0.947	1	3.91E-01	5.45E-01
**124**	EPM2AIP1	0.862	0.917	0.957	1	4.23E-01	5.71E-01
**125**	TDRD9	1.615	1.329	1.054	1	4.25E-01	5.72E-01
**126**	AP3M2	0.815	0.871	0.951	1	4.51E-01	5.93E-01
**127**	IL17RC	1.119	1.100	1.050	1	4.55E-01	5.95E-01
**128**	ELANE	1.310	1.413	1.048	1	4.63E-01	6.01E-01
**129**	AGTRAP	1.251	1.149	0.957	0	4.87E-01	6.19E-01
**130**	OLFM4	2.109	1.948	0.956	0	4.99E-01	6.28E-01
**131**	HIST1H1B	1.128	1.090	0.960	0	4.99E-01	6.28E-01
**132**	BCL11B	0.828	0.865	0.960	1	5.02E-01	6.30E-01
**133**	PSMB3	1.178	1.110	0.963	0	5.27E-01	6.48E-01
**134**	ZNF789	0.877	0.913	0.964	1	5.28E-01	6.49E-01
**135**	DDX27	0.860	0.926	1.034	0	5.83E-01	6.88E-01
**136**	PLIN3	1.233	1.133	1.024	1	7.08E-01	7.82E-01
**137**	RTTN	0.732	0.894	0.980	1	7.65E-01	8.24E-01
**138**	RNASE3	1.781	1.773	1.017	1	7.93E-01	8.45E-01
**139**	RETN	1.307	1.197	0.985	0	8.10E-01	8.58E-01
**140**	NOL9	0.887	0.902	0.985	1	8.18E-01	8.64E-01
**141**	S100A6	1.158	1.164	1.013	1	8.19E-01	8.65E-01
**142**	CEP95	0.832	0.872	1.013	0	8.20E-01	8.66E-01
**143**	CHIT1	1.242	1.268	0.986	0	8.27E-01	8.71E-01
**144**	P2RY8	0.909	0.924	0.991	1	8.87E-01	9.16E-01
**145**	TIAM1	0.881	0.911	0.992	1	8.99E-01	9.25E-01
**146**	PCOLCE2	1.185	1.209	0.997	0	9.59E-01	9.69E-01
**147**	DYNC2H1	0.805	0.801	0.997	1	9.63E-01	9.72E-01
**148**	CEBPA	1.324	1.144	1.001	1	9.83E-01	9.87E-01
**149**	DEFA4	2.102	1.703	1.001	1	9.85E-01	9.89E-01
**150**	ZDHHC12	1.174	1.127	1.000	0	1.00E+00	1.00E+00

Table S9. List of 21 genes that are significantly differentially expressed (q2 = 0.0084) with consistent sign of fold change in both the GSK-HiTDIP and Janssen-BRC studies when not correcting for anxiety. This list was defined using a Bayesian rule for finding the largest P-value threshold associated with twice as many differentially expressed genes in both studies as would be expected by random coincidence (implemented in sdef software; Blangiardo et al [14]). Genes are ranked according to the HiTDIP fold-change (FC). The other columns indicate: genes that were also reported within the list of 165 genes in Table S4 (column "In list of 165"), genes that were measured in the NESDA study (column "in NESDA") and genes which were statistically significant at 5%, 10% and 20% FDR thresholds in the NESDA study (columns "NESDA 5%", "NESDA 10%" and "NESDA 20%", respectively). The last column of the table provides the fold-changes of the genes within the NESDA study.

Rank	Symbol	HiTDIP FC	BRC FC	In list of 165	In NESDA	NESDA 5%	NESDA 10%	NESDA 20%	NESDA FC
**1**	MMP8	2.215	1.566	1	1	0	0	0	1.066
**2**	HP	1.696	1.419	1	1	0	0	0	1.129
**3**	NRG1	1.449	1.389	1	1	0	1	1	1.237
**4**	RNASE1	1.397	1.354	1	1	0	0	0	0.913
**5**	SLPI	1.394	1.463	1	1	0	1	1	1.237
**6**	CAMP	1.370	1.374	1	1	0	0	1	1.162
**7**	S100A12	1.357	1.212	1	1	0	0	1	1.142
**8**	PGLYRP1	1.324	1.374	1	1	1	1	1	1.254
**9**	MGST1	1.250	1.145	1	1	0	0	0	1.142
**10**	LOC100507507	1.191	1.115	0	0	0	0	0	NA
**11**	S100A9	1.127	1.107	1	1	0	0	1	1.170
**12**	AHNAK	0.920	0.935	0	1	0	0	0	0.969
**13**	TBP	0.906	0.934	1	1	0	0	0	0.921
**14**	IFT20	0.898	0.914	0	1	0	0	1	1.148
**15**	IL10RA	0.893	0.935	0	1	0	1	1	0.829
**16**	MDN1	0.882	0.913	0	1	0	0	0	0.960
**17**	CBY1	0.877	0.930	1	1	0	0	0	0.947
**18**	SRSF6	0.875	0.914	1	1	0	0	1	0.856
**19**	TBCE	0.862	0.916	0	1	0	0	0	0.922
**20**	NFATC2	0.857	0.877	1	1	1	1	1	0.799
**21**	THOC1	0.853	0.890	1	1	0	0	1	0.877

Table S10. Enrichment analysis of the MDD-21 consensus set of genes that were differentially over-expressed (UP) or under-expressed (DOWN) in MDD patients compared to healthy controls in both GSK-HiTDiP and Janssen-BRC studies when not correcting for anxiety. The top 15 gene ontology (GO) terms for Biological Processes (BP) are ranked according to their P-values by Fisher's exact test for significant enrichment. Bonferroni correction specifies a P-value threshold of P = 4.94 x 10-6 to achieve significance at q < 0.05. Such a correction is well-known to be too stringent given that many of the GO terms are correlated; however, the top 8 terms were significantly enriched in the over-expressed UP gene set after Bonferroni correction.

Over-expressed UP						
**Rank**	**GO.ID**	**Term**	**Annotated**	**Significant**	**Expected**	**Fisher**
1	GO:0042742	defense response to bacterium	183	6	0.13	7.7e-10
2	GO:0009617	response to bacterium	442	7	0.31	2.7e-09
3	GO:0043207	response to external biotic stimulus	782	7	0.54	1.4e-07
4	GO:0051707	response to other organism	782	7	0.54	1.4e-07
5	GO:0009607	response to biotic stimulus	813	7	0.56	1.8e-07
6	GO:0098542	defense response to other organism	465	6	0.32	2.0e-07
7	GO:0050832	defense response to fungus	30	3	0.02	9.6e-07
8	GO:0009620	response to fungus	43	3	0.03	2.9e-06
9	GO:0009605	response to external stimulus	2346	8	1.62	1.6e-05
10	GO:0045087	innate immune response	980	6	0.68	1.6e-05
11	GO:0006952	defense response	1602	7	1.11	1.8e-05
12	GO:0006954	inflammatory response	579	5	0.4	2.2e-05
13	GO:0044110	growth involved in symbiotic interaction	22	2	0.02	9.9e-05
14	GO:0044116	growth of symbiont involved in interacti...	22	2	0.02	9.9e-05
15	GO:0051704	multi-organism process	2122	7	1.47	0.00012
**Under-expressed (DOWN)**						
**Rank**	**GO.ID**	**Term**	**Annotated**	**Significant**	**Expected**	**Fisher**
1	GO:0051146	striated muscle cell differentiation	228	3	0.16	0.00043
2	GO:0045934	negative regulation of nucleobase-contai...	1241	5	0.86	0.00081
3	GO:0006406	mRNA export from nucleus	63	2	0.04	0.00082
4	GO:0071427	mRNA-containing ribonucleoprotein comple...	63	2	0.04	0.00082
5	GO:0051172	negative regulation of nitrogen compound...	1335	5	0.92	0.00113
6	GO:0071426	ribonucleoprotein complex export from nu...	74	2	0.05	0.00113
7	GO:0006405	RNA export from nucleus	75	2	0.05	0.00117
8	GO:0071166	ribonucleoprotein complex localization	76	2	0.05	0.0012
9	GO:0043484	regulation of RNA splicing	79	2	0.05	0.00129
10	GO:0008380	RNA splicing	335	3	0.23	0.00131
11	GO:0042692	muscle cell differentiation	335	3	0.23	0.00131
12	GO:0006353	DNA-templated transcription, termination	82	2	0.06	0.00139
13	GO:0055007	cardiac muscle cell differentiation	85	2	0.06	0.00149
14	GO:0050864	regulation of B cell activation	98	2	0.07	0.00198
15	GO:0035051	cardiocyte differentiation	103	2	0.07	0.00218

Table S11. Gene transcriptional network modules were tested for case-control differences in eigengene expression. The 17 module eigengenes were extracted from the normative transcriptional network estimated from the control groups pooled over both studies. The gene expression data for each individual participant, whether a case or control, was projected onto each eigengene to summarise their expression of modular genes. Two-tailed t-tests were performed to compare the mean eigengene score between groups for each module: positive *t*-statistics indicate that "on average" over all genes in each module there was relative over-expression in MDD cases compared to controls; negative *t*-statistics indicate that "on average" over all genes in each module there was relative under-expression in MDD cases compared to controls. There were significant case-control differences in eigengene expression for four modules, as shown below: module colours and functional labels correspond to **Figure 2**.

Module	N Genes	t	df	P-value	Top Enriched Terms
Pink	504	-3.05	349.77	**0.002**	Translation termination/translation initiation/SRP-dependent cotranslational protein targeting to membrane (+ adaptive immune response)
Yellow	2138	-2.45	334.41	**0.015**	Inflammatory response/myeloid cell activation involved in immune response/phagocytosis
Tan	130	-2.26	356.77	**0.025**	No significant enrichment
Red	748	2.17	356.62	**0.031**	Inflammatory response/ positive regulation of intracellular signal transduction/positive regulation of cellular processes
Cyan	106	1.85	325.49	0.066	No significant enrichment
Purple	163	-1.71	318.41	0.088	Type I interferon signalling pathway
Black	538	-1.63	349.84	0.105	Erythrocyte development
Green	1381	1.48	336.37	0.140	No significant enrichment
Magenta	205	1.35	317.94	0.177	SRP-dependent cotranslational protein targeting to membrane/ translational termination
Midnight blue	91	1.30	355.20	0.195	SRP-dependent cotranslational protein targeting to membrane/translational initiation and termination
Brown	2235	1.14	350.39	0.256	rRNA processing/translation/respiratory electron transport chain
Turquoise	4299	-1.06	328.14	0.291	Regulation of RNA metabolic process/regulation of gene expression/regulation of macromolecule biosynthesis
Blue	3694	-1.02	321.20	0.310	No significant enrichment
Grey	2235	0.91	299.78	0.365	No significant enrichment
Salmon	115	0.51	285.91	0.607	No significant enrichment
Green yellow	134	-0.07	341.74	0.947	Platelet activation/platelet degranulation
Light cyan	79	-0.01	350.61	0.996	No significant enrichment

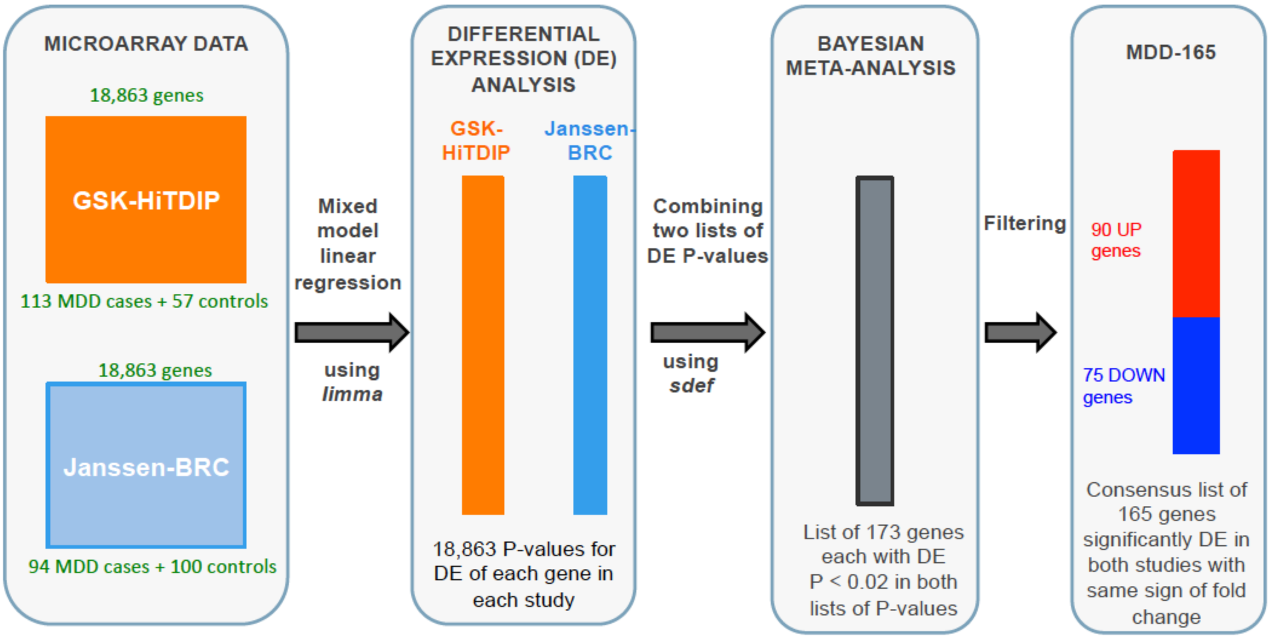

Figure S1. Schematic of study design and analysis

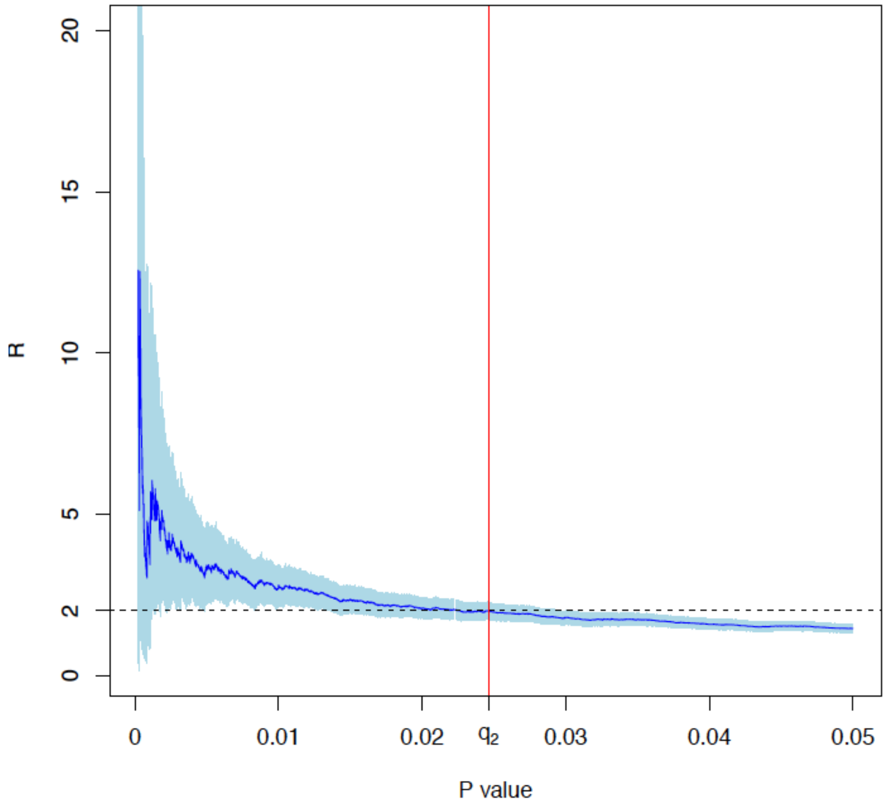

Figure S2. Bayesian analysis of genes differentially expressed in patients with MDD compared to healthy controls in both GSK-HiTDiP and Janssen-BRC studies. The ratio, *R*, between the observed and randomly expected number of genes differentially expressed in both studies (*y*-axis; dark blue line = median, light blue area = 95% credibility interval) increases and becomes more variable as the *P*-value threshold (x-axis) used to define significant case-control differences in each study becomes smaller or more conservative. The threshold q_2 = 0.025 (red line) is the *P*-value at which the ratio *R(q)* = 2 (dotted line), i.e., the number of genes differentially expressed in both studies is twice the number expected by chance under the null hypothesis. It was previously shown by an extensive simulation that this choice of threshold gives a good compromise between specificity and sensitivity (13, 14).

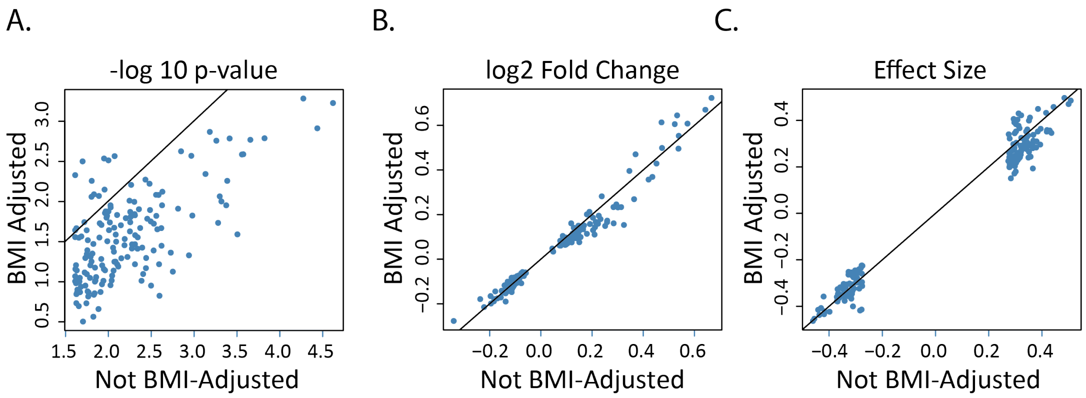

Figure S3. Statistical correction for effects of body mass index (BMI) on significance and effect size of case-control differences in the MDD-165 consensus gene set. A. Comparison of –log10 *P*-values for BMI-adjusted vs. BMI-unadjusted (“raw”) gene expression data. B. Comparison of log2 fold change difference between cases and controls for BMI-adjusted vs. BMI-unadjusted gene expression data. C. Comparison of effect sizes of case-control differences, for BMI-adjusted vs. BMI-unadjusted gene expression data.

Supplementary References

1. Domenici E, Willé DR, Tozzi F, Prokopenko I, Miller S, McKeown A, et al. (2010): Plasma protein biomarkers for depression and schizophrenia by multi analyte profiling of case-control collections. . *PLoS ONE*. 5:e9166.
2. Muglia P, Tozzi F, Galwey NW, Francks C, Upmanyu R, Kong XQ, et al. (2010): Genome-wide association study of recurrent major depressive disorder in two European case–control cohorts. *Molecular Psychiatry*. 15:589-601.
3. Tilahun A, Lin D, Shkedy Z, Geys H, Alonso A, Peeters P, et al. (2010): Genomic biomarkers for depression: Feature-specific and joint biomarkers. *Statistics in Biopharmaceutical Research* 2:419-434.
4. Zoon H, Veth C, Arns M, Drinkenburg W, Talloen W, Peeters P, et al. (2013): EEG alpha power as an intermediate measure between brain-derived neurotrophic factor Val66Met and depression severity in patients with major depressive disorder. *J Clin Neurophys* 30:261-267.
5. Liu Y, Yieh L, Yang T, Drinkenburg W, Peeters P, Steckler T, et al. (2016): Metabolomic biosignature differentiates melancholic depressive patients from healthy controls. *BMC Genomics*. 17:669.
6. Wing JK, Babor T, Brugha T, Burke J, Cooper JE, Giel R, et al. (1990): SCAN: Schedules for Clinical Assessment in Neuropsychiatry. *Arch Gen Psychiatry*. 47:589-593.
7. Wittchen HU, Höfler M, Gander F, Pfister H, Storz S, Üstün B, et al. (1999): Screening for mental disorders: performance of the Composite International Diagnostic–Screener (CID–S). *International Journal of Methods in Psychiatric Research*. 8:59-70.
8. Parker G, Hadzi-Pavlovic D, Boyce P, Wilhelm K, Brodaty H, Mitchell P, et al. (1990): Classifying depression by mental state signs. *British Journal Psychiatry*. 157:55-65.
9. Gautier L, Cope L, Bolstad BM, Irizarry RA (2004): affy—analysis of Affymetrix GeneChip data at the probe level *Bioinformatics*. 20:307-315.
10. Kauffmann A, Gentleman R, Huber W (2009): arrayQualityMetrics—a bioconductor package for quality assessment of microarray data. . *Bioinformatics*. 25:415-416.
11. Ritchie ME, Phipson B, Wu D, Hu Y, Law CW, Shi W, et al. (2015): limma powers differential expression analyses for RNA-sequencing and microarray studies. *Nucleic Acids Research*. gkv007.
12. Li Q, Birkbak NJ, Gyorffy B, Szallasi Z, Eklund AC (2011): Jetset: selecting the optimal microarray probe set to represent a gene. *BMC Bioinformatics*. 12:1.
13. Blangiardo M, Richardson S (2007): Statistical tools for synthesizing lists of differentially expressed features in related experiments. *Genome Biology*. 8:R54.
14. Blangiardo M, Cassese A, Richardson S (2010): sdef: an R package to synthesize lists of significant features in related experiments. . *BMC Bioinformatics*. 11:270.
15. Benjamini Y, Hochberg Y (1995): Controlling the false discovery rate: a practical and powerful approach to multiple testing. *Journal of the Royal Statistical Society Series B (Methodological)*. 57:289-300.
16. Langfelder P, Horvath S (2008): WGCNA: an R package for weighted correlation network analysis. *BMC Bioinformatics*. 9:1.
17. Freeman TC, Goldovsky L, Brosch M, Van Dongen S, Mazière P, Grocock RJ, et al. (2007): Construction, visualisation, and clustering of transcription networks from microarray expression data. *PLoS Comput Biol*. 3:e206.

18. Theocharidis A, van Dongen S, Enright A, Freeman T (2009): Network visualization and analysis of gene expression data using BioLayout Express (3D). *Nature Protocols*. 4:1535-1550.

19. Jansen R, Penninx BWJH, Madar V, Xia K, Milaneschi Y, Hottenga JJ, et al. (2016): Gene expression in major depressive disorder. *Molecular Psychiatry*. 21:444.

20. Mostafavi S, Battle A, Zhu X, Potash JB, Weissman MM, Shi J, et al. (2014): Type I interferon signaling genes in recurrent major depression: increased expression detected by whole-blood RNA sequencing. *Molecular Psychiatry*. 19:1267-1274.

21. Spijker S, Van Zanten JS, De Jong S, Penninx BW, van Dyck R, Zitman FG, et al. (2010): Stimulated gene expression profiles as a blood marker of major depressive disorder. *Biological Psychiatry*. 68:179-186.

22. Savitz J, Frank M, Victor T, Marino J, McJinney B, Teague T, et al. (2013): Inflammatory, apoptotic, and neurological disease-related genes are differentially expressed in currently depressed patients with mood disorders and correlate with morphometric and functional imaging abnormalities. *Brain Behavior & Immunity*. 31:161-171.

23. Segman RH, Goltser-Dubner T, Weiner I, Canetti L, Galili-Weisstub E, Milwidsky A, et al. (2010): Blood mononuclear cell gene expression signature of postpartum depression. *Molecular Psychiatry*. 15:93-100.

24. Yi Z, Li Z, Yu S, Yuan C, Hong W, Wang Z, et al. (2012): Blood-based gene expression profiles models for classification of subsyndromal symptomatic depression and major depressive disorder *PLoS ONE*. 7:p.e31283.

25. Glahn DC, Curran JE, Winkler AM, Carless MA, Kent JW, Charlesworth JC, et al. (2012): High dimensional endophenotype ranking in the search for major depression risk genes. *Biological Psychiatry*. 71:6-14.
